# Multifunctional sulfonium-based treatment for perovskite solar cells with less than 1% efficiency loss over 4,500-h operational stability tests

**DOI:** 10.1038/s41560-023-01421-6

**Published:** 2024-01-04

**Authors:** Jiajia Suo, Bowen Yang, Edoardo Mosconi, Dmitry Bogachuk, Tiarnan A. S. Doherty, Kyle Frohna, Dominik J. Kubicki, Fan Fu, YeonJu Kim, Oussama Er-Raji, Tiankai Zhang, Lorenzo Baldinelli, Lukas Wagner, Ayodhya N. Tiwari, Feng Gao, Andreas Hinsch, Samuel D. Stranks, Filippo De Angelis, Anders Hagfeldt

**Affiliations:** 1 Department of Chemistry–Ångström Laboratory, Uppsala University, Uppsala, Sweden; 2 Laboratory of Photomolecular Science, Institute of Chemical Sciences and Engineering, School of Basic Sciences, Ecole Polytechnique Fédérale de Lausanne, Lausanne, Switzerland; 3 Computational Laboratory for Hybrid/Organic Photovoltaics (CLHYO), Istituto CNR di Scienze e Tecnologie Chimiche ‘Giulio Natta’ (CNR-SCITEC), Perugia, Italy; 4 Fraunhofer Institute for Solar Energy Systems ISE, Freiburg, Germany; 5 Department of Sustainable Systems Engineering (INATECH), Albert-Ludwigs-Universität Freiburg, Freiburg, Germany; 6 Solarlab Aiko Europe GmbH, Freiburg, Germany; 7 Department of Chemical Engineering and Biotechnology, University of Cambridge, Cambridge, UK; 8 Department of Materials Science and Metallurgy, University of Cambridge, Cambridge, UK; 9 Cavendish Laboratory, Department of Physics, University of Cambridge, Cambridge, UK; 10 Department of Physics, University of Warwick, Coventry, UK; 11 Laboratory for Thin Films and Photovoltaics, Empa−Swiss Federal Laboratories for Materials Science and Technology, Duebendorf, Switzerland; 12 Laboratory for Molecular Engineering of Optoelectronic Nanomaterials, Institute of Chemical Sciences and Engineering, School of Basic Sciences, Ecole Polytechnique Fédérale de Lausanne, Lausanne, Switzerland; 13 Department of Physics, Chemistry and Biology (IFM), Linköping University, Linköping, Sweden; 14 Department of Chemistry, Biology and Biotechnology, University of Perugia, Perugia, Italy; 15 Physics of Solar Energy Conversion Group, Department of Physics, Philipps-University Marburg, Marburg, Germany; 16 Department of Natural Sciences and Mathematics, College of Sciences and Human Studies, Prince Mohammad Bin Fahd University, Dhahran, Saudi Arabia; 17 SKKU Institute of Energy Science and Technology (SIEST), Sungkyunkwan University, Suwon, Korea; 18 Present Address: School of Chemistry, University of Birmingham, Edgbaston, UK

**Keywords:** Solar cells, Energy

## Abstract

The stabilization of grain boundaries and surfaces of the perovskite layer is critical to extend the durability of perovskite solar cells. Here we introduced a sulfonium-based molecule, dimethylphenethylsulfonium iodide (DMPESI), for the post-deposition treatment of formamidinium lead iodide perovskite films. The treated films show improved stability upon light soaking and remains in the black *α* phase after two years ageing under ambient condition without encapsulation. The DMPESI-treated perovskite solar cells show less than 1% performance loss after more than 4,500 h at maximum power point tracking, yielding a theoretical *T*_80_ of over nine years under continuous 1-sun illumination. The solar cells also display less than 5% power conversion efficiency drops under various ageing conditions, including 100 thermal cycles between 25 °C and 85 °C and an 1,050-h damp heat test.

## Abstract (web summary)

Suo et al. show that sulfonium-based molecules afford formamidinium lead iodide perovskites protection against environmental stress factors, improved phase stability and solar cells retaining efficiency over 4,500-h operational stability tests.

## Main

Efficiency, stability and scalability are the most important factors on the route towards commercialization of perovskite solar cells (PSCs). Remarkable certified power conversion efficiencies (PCEs) have achieved 25.7% on single-junction PSCs and 32.5% on perovskite-silicon tandem solar cells^1^. Recently, scalability has also been effectively managed by modifying the charge transport layers and interfacial treatments with up-scalable deposition techniques, which enabled certified PCEs of 23.1, 22.7 and 20.5% on 1, 24 and 64 cm^2^ cells and mini-modules, respectively^2–4^. Although stability has been notably improved, it still remains the most critical limitation for commercializing state-of-the-art highly efficient perovskite-based photovoltaics and further improvement is critically required^5–7^.

Both performance loss and degradation of PSCs are initiated at grain boundaries and interfaces, where defects and mobile ions tend to accumulate under external stress, such as continuous illumination, humid environment and elevated temperature^8–12^. Therefore, suppressing surface defects along with inhibiting mobile ion migration is critical to achieve PSCs long-term stability. Different molecular species have been explored to suppress defect formation and ion migration either in the perovskite bulk or at the adjacent interfaces, with the ultimate goal of achieving stable PSCs. These species include ammonium-based salts, small organic molecules, polymers and other passivation agents including inorganic salts^13–16^. A category of aprotic sulfonium-based molecules shows great potential in stabilizing PSCs, however, it remains largely unexplored^17^. The innovation of utilizing such material to stabilize PSCs are driven by the unique geometries with largely strengthened chemical and humidity stability provided by the sulfonium-based cations. For example, a 1D perovskite ((CH_3_)_3_SPbI_3_) based on the trimethylsulfonium cation has been synthesized, which exhibits elevated chemical stability^18^. Similarly, a fully alkylated butyldimethylsulfonium iodide was introduced into perovskite, which exhibited a pronounced enhancement in device stability under humid conditions^19^. However, the poor solubility of these aliphatic sulfonium salts^18–21^ in low polar solvents (such as isopropanol and chloroform) limits their application for surface treatment.

**Fig. 1:**
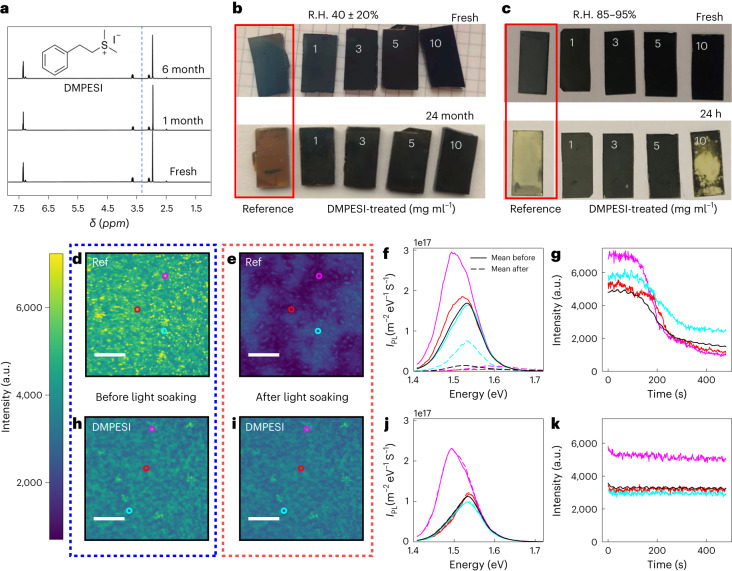
Stability of DMPESI molecule and treated perovskite films under moisture and light-soaking conditions. **a**, ^1^H NMR spectra of DMPESI in anhydrous DMSO-d6 after six months of storage in ambient air. The H_2_O peak in DMSO-d6 is at 3.3 ppm (vertical dashed blue line): the signal is absent in the samples. The chemical structure of DMPESI is shown. **b**, Photographs of fresh and 24-month aged unencapsulated perovskite film (1.0 cm by 2.0 cm) without and with DMPESI treatment of different concentrations (from left to right: reference, 1 mg ml^−1^, 3 mg ml^−1^, 5 mg ml^−1^, 10 mg ml^−1^) in ambient air with R.H. 40 ± 20%. **c**, Photographs of fresh and 24 h aged unencapsulated perovskite film (1.0 cm by 2.0 cm) without and with DMPESI treatment of different concentrations (from left to right: reference, 1 mg ml^−1^, 3 mg ml^−1^, 5 mg ml^−1^, 10 mg ml^−1^) in high humid condition of R.H. 85∼95%. (**d**,**e**,**h**,**i**) Hyperspectral photoluminescence microscopy of FAPbI_3_ reference and DMPESI-treated films, scale bar = 15 μm. **d**,**e**, Photoluminescence (PL) map of reference FAPbI_3_ film before (**d**) and after (**e**) 10 minutes of light soaking in air at 1-sun illumination intensity with a 405 nm continuous wave laser. **f**, PL spectra before (solid lines) and after (dashed lines) light from the coloured points marked in panels **d** and **e**. The mean PL spectrum of the region is marked in black. *I*_P__L_ is PL intensity. **g**, Photoluminescence intensity changes with light soaking of the marked regions in **d** and the average of the image (black). **h**,**i**, Photoluminescence intensity map of DMPESI-treated film before (**h**) and after (**i**) 10 minutes of light soaking (1-sun equivalent). **j**, PL spectra before (solid lines) and after (dashed lines) light soaking of the marked regions in **h** and **i**. **k**, PL intensity changes with light soaking of the marked regions in **h** and the average of the image (black).

In this work, we synthesized and introduced an unexplored dimethylphenethylsulfonium cation and its iodide salt, DMPESI (Fig. 1a, inset), endowed with an aromatic moiety, and used it as a surface passivation agent on FAPbI_3_-based PSCs. We pose the aromatic nature of the ligand induces a close packing on the perovskite surface compared with aliphatic chains. The results indicate that one-step perovskite surface treatment with DMPESI effectively achieves a series of benefits: it passivates surface defects, prolonging charge-carrier lifetime and suppressing non-radiative recombination, resulting in superior PSCs performance; it enhances thermodynamic stability of black FAPbI_3_, preventing phase transition to *δ* phase; it induces formation of a compact and ultra-hydrophobic capping layer on top of the perovskite surface, which inhibits ion migration and impedes ambient-induced degradation mechanisms. Consequently, the DMPESI-treated FAPbI_3_ film exhibits much improved stability upon light soaking and remains in the black phase after two years ageing under ambient condition. Moreover, the resulting high-efficiency (PCE > 23%) PSCs exhibit long-term stability, showing: a negligible PCE drop (less than 1%) after more than 4,500 h maximum power point tracking, which is among the most operationally stable PSCs reported so far; they retain more than 96% of initial performance without encapsulation at 60 °C with relative humidity (R.H.) < 30% after 400 h ageing; the encapsulated devices retain over 98% of their initial performance under thermal cycling, following the International Summit on Organic Photovoltaic Stability (ISOS) stability protocol; the encapsulated devices successfully pass the damp heat test (85 °C at 85% R.H.) for over 1,000 h with less than 5% loss in PCE. Such excellent durability of our DMPESI-treated perovskite film and the PSC devices provides a strong indication that perovskite thin films have the potential to fulfil industrial protocols and compete with Si-based solar cells for further large-scale and industrial productions.

## Perovskite film stability under moisture and light soaking

We started by evaluating the stability of the DMPESI salt by storing it in ambient air with R.H. of 40 ± 20% and tracking it using ^1^H nuclear magnetic resonance (^1^H NMR) measurement. As evident from the spectra in Fig. 1a, no H_2_O peak is detected after six months of storage. Such low water uptake property of aprotic DMPESI is expected to improve the hydrophobicity of the perovskite film after surface treatment, and thus the device stability, especially under humid conditions. The hydrophobicity of DMPES^+^ against a related ammonium-based phenethylammonium cation (PEA^+^) is confirmed by mapping the electrostatic potential of both cations, as derived by density function theory (DFT) calculations (Supplementary Note 1), showing a high polarity region close to the ammonium group in PEA^+^, which is lacking in DMPES^+^. Similarly, solvation free energies and water interaction energies calculated for both cations clearly show a reduced tendency to bind water for DMPES^+^ against PEA^+^.

We deposit a layer of 1–10 mg ml^−1^ of DMPESI on top of a 3D perovskite film, where formamidinium lead triiodide (FAPbI_3_) is selected, owing to its favourable bandgap suitable for high-performance PSCs^22,23^. The morphologies of the perovskite films are presented in Supplementary Fig. 2. A dense and pinhole-free polycrystalline perovskite film with micrometre-sized grains becomes more uniform upon surface treatment even with low amount (1 mg ml^−1^) of DMPESI. As the concentration of DMPESI increases to 3 mg ml^−1^ and 5 mg ml^−1^, a uniform and smooth surface is still observed, however, new species also appear. Notably, when the concentration of DMPESI further increases to 10 mg ml^−1^, some cracks are observed on the perovskite surface, which might be due to the aggregation of the excess DMPESI.

Contact angle measurements of water droplets on the surface were carried out to evaluate the moisture stability of the perovskite film (Supplementary Fig. 3). A pronounced increase in contact angle is observed from 55.8° of the reference perovskite to around 90° of the DMPESI-treated films, regardless of concentration, suggesting a more hydrophobic surface upon treatment. Then we exposed the perovskite films to ambient air. The reference film becomes brownish after exposure for ten days. In contrast, the perovskite films treated with 3 mg ml^−1^ and 5 mg ml^−1^ DMPESI remain in pure black phase under the same condition even after 24 months (Fig. 1b and Supplementary Fig. 4), as evidenced by the X-ray diffraction (XRD) patterns of the aged samples in Supplementary Fig. 5. We further expose the perovskite films to a high R.H. of 85–95%. Photographs of the films are shown in Fig. 1c and Supplementary Fig. 6 and their corresponding XRD patterns are monitored in Supplementary Fig. 7. After exposure for 24 h, the reference perovskite film experiences a rapid transformation from black phase to yellow phase FAPbI_3_. In addition, the peak at 13.9° for *α* (110) exhibits dramatic decrease in intensity, suggesting an inferior perovskite crystallinity with larger disordered regions between grains^24^. However, in sharp contrast, films treated with moderate quantities (1 mg ml^−1^, 3 mg ml^−1^ and 5 mg ml^−1^) of DMPESI retain in the black phase FAPbI_3_. Interestingly, the perovskite film treated with excessive DMPESI (10 mg ml^−1^) displays aggravated phase instability. We attribute this to the poor surface coverage caused by the aggregation of the excess DMPESI, supported by the observed scanning electron microscope (SEM) image in Supplementary Fig. 2, which could induce degradation pathways for moisture from external atmosphere to penetrate into the perovskite bulk, leading to an accelerated degradation process.

To further probe the light-soaking stability of the reference and DMPESI-treated (3 mg ml^−1^) films, we performed wide-field, hyperspectral PL mapping of both samples in ambient conditions. Hyperspectral PL maps (Fig. 1d,h) display similar spatial PL distributions with comparable emissions intensities and peak positions and an additional small red-shifted PL. The DMPESI-treated sample shows enhanced low energy emission around the edges of the grains in comparison with the reference sample (Supplementary Fig. 8). The samples were then light soaked for 10 minutes with 1-sun illumination intensity under ambient conditions. The corresponding maps of the same areas of the reference and treated samples are shown in Fig. 1e,i, respectively. Notably, the intensity of the reference sample drops dramatically during illumination and distinct regions tens of microns in size with higher or lower PL emerge (Supplementary Video 1). As shown in Fig. 1f and Supplementary Fig. 9, the regions that lose the most intensity also display a dramatic PL blue shift of ∼75 meV and the emergence of a second peak at much higher energy close to 2 eV. This suggests that the reference FAPbI_3_ phase is highly unstable and over the course of ten minutes of light soaking, multiple new phases/compositions emerge, negatively affecting the optoelectronic quality of the material. The regions that retain the most PL intensity simply drop in intensity without any peak shift. The instability of the reference sample is further highlighted in Fig. 1g, which plots the integrated PL intensity at different points on the sample. By contrast, the DMPESI-treated sample displays remarkably different behaviour. The treated sample exhibits effectively no transient behaviour over the same time period, with negligible changes to the PL intensity, spatial distribution or spectra as shown in Fig. 1i–k and Supplementary Video 2. These results collectively show that the DMPESI treatment has turned an intrinsically unstable material into a much more stable one both macroscopically and microscopically.

## Atomistic mechanism with perovskite

**Fig. 2:**
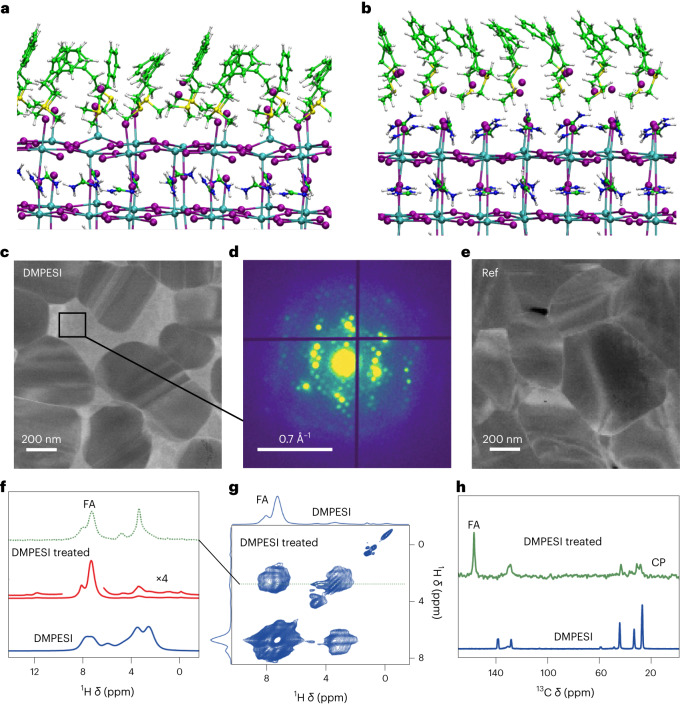
Microstructure of the perovskite films and interactions between DMPESI and FAPbI_3_. **a**,**b**, DMPESI/FAPbI_3_ perovskite full-coverage interface DFT models at PbI_2_- (**a**) and FAI-terminated (**b**) surfaces. The following colour code is used for the atomic representations: purple, I; cyan, Pb; blue, N; yellow, S; green, C; white, H. **c**, Annular dark-field image reconstructed from SED data of DMPESI-treated perovskite film. **d**, The ED patterns extracted from intragranular region are shown. **e**, Annular dark-field image reconstructed from SED data of reference film. Atomic-level interaction between FAPbI_3_ and DMPESI from solid-state NMR. **f**, Quantitative ^1^H MAS NMR spectra of DMPESI (blue) and DMPESI-treated FAPbI_3_ prepared as spin-coated thin films (red). **g,** ^1^H–^1^H spin-diffusion spectrum of DMPESI-treated FAPbI_3_ prepared as spin-coated thin films (50 kHz MAS, 23 T). The horizontal cross section at 3.4 ppm (dashed line) is shown in green. The spectrum of DMPESI is shown in green in panel **f**. **h**, ^1^H–^13^C cross polarization MAS spectra of DMPESI and DMPESI-treated FAPbI_3_ prepared as a drop-cast film. FA is formamidinium, CP is cross polarization, ×4 is 4 times intensity of the signal.

To explore the reasons underlying stability of the DMPESI-treated perovskite film, we carried out DFT calculations of absorbed DMPESI and phenylethylammonium iodide (PEAI) layers on PbI_2_- and formamidinium iodide (FAI)-terminated perovskite surfaces at various surface coverage, as shown in Fig. 2a,b for the full-coverage systems^25,26^ (detailed approach in the Supplementary Information). The interaction of a single DMPESI or PEAI unit on the PbI_2_-terminated surface provides us with the strength of the salt/perovskite surface, devoid of intermolecular packing interactions (Supplementary Fig. 10c–e). Supplementary Table 2 shows that the dissociative adsorption of the iodide salts, with formation of surface Pb–I bond, is strongly favoured in DMPESI vs PEAI (2.09 vs 1.74 eV) and effectively supersedes surface FAI bonding (1.61 eV). The strong interaction energy is a prerequisite to effectively prevent surface dissolution during device operation. At half coverage, DMPESI binding is still favoured over PEAI (1.93 vs 1.71 eV per molecule) though PEAI forms a slightly more compact layer (Supplementary Fig. 10f–g and Supplementary Table 2). Surprisingly, at full coverage, DMPESI preferentially forms an overlayer (about half of interacting molecules do not bind the perovskite surface) whereas PEAI forms an ordered monolayer (Supplementary Fig. 10h,i), with comparable interaction energies dominated by intermolecular interactions. The absence of directional hydrogen bond in the sulfonium-based DMPESI does not induce an ordered molecular orientation as it is found in the PEAI salt endowed with R-NH_3_^+^ groups. On a fully FAI-terminated perovskite surface, DMPESI also forms a strong protective layer with enhanced interaction energy (1.69 vs 1.28 eV) dominated by dispersion forces (Supplementary Fig. 10l,m). Thus DMPES^+^ can effectively assist the encapsulation of mobile iodide anions via the sulfonium groups and the phenyl rings in the DMPESI framework. This encapsulation could also reduce the presence of interstitial iodine defects at the PbI_2_-terminated surface, which are demonstrated to be a hole-trapping centre^27^. Moreover, the electronic properties of the passivated slab with a DMPESI layer show a reduction of the surface traps with respect to the bare PbI_2_-terminated surface^28^, even if only half of the exposed surface Pb atoms are passivated by iodide, as shown in Supplementary Fig. 11. The interlayer in this case does not introduce notable changes on the electronic structure with respect to the bare FAI-terminated surface (Supplementary Fig. 11). Both DMPESI and PEAI are also able to effectively passivate surface FAI vacancies (Supplementary Fig. 10n,o).

Overall, our analysis indicates the salient features of DMPESI/perovskite interaction that make this salt different from standard PEAI. Basically, DMPESI is able to strongly bind the perovskite surface forming a surface hydrophobic layer. The strong surface interaction stabilizes the perovskite layer probably locking it into the black phase. At high coverage, however, the competition between surface/molecule and intermolecular interactions induces the formation of a DMPESI overlayer, which may limit charge extraction and to some extent prevent full surface passivation.

Scanning electron diffraction (SED) measurements have been carried out to understand the impact of DMPESI on the microstructure of the perovskite film^29^. An annular dark-field (ADF) image reconstructed from SED data reveals variation in the diffraction contrast across the perovskite film with brighter regions appearing between distinct grains, as shown in Fig. 2c. Examining the SED pattern extracted from one of the grains reveals a perovskite grain oriented near the [001]_c_ zone axis. The presence of superstructure reflections, forbidden from appearing in the cubic perovskite phase (white arrows in Supplementary Fig. 12b), indicates the presence of octahedral tilting, which has been shown to frustrate the phase transition from photoactive FA-rich perovskites to the photoinactive *δ* phase^30^. Examining the SED pattern extracted from the intragranular region—Fig. 2d (in comparison with reference film in Fig. 2e)—indicates the presence of an additional crystalline phase, which can also be observed by the emission morphologies, shown in Supplementary Fig. 8. Combining with the results obtained from XRD and grazing-incidence wide angle X-ray scattering (GIWAXS), shown in Supplementary Fig. 13, we suspect the short diffraction vectors can most likely be attributed to low-dimensional perovskite structures formed between the grains of the 3D FAPbI_3_ bulk upon DMPESI treatment. Octahedral tilting is also observed in the reference perovskite film, as indicated in Supplementary Fig. 12d,e. This probably arises from the alloying trace amount of MA^+^ on the A site with FA^+^ and imbues a degree of stability (that is, the reference film does not turn entirely to *δ* phase immediately), although is not sufficient to firmly stabilize the perovskite film. The synergistic effect of the DMPESI at the grain boundaries and the structural octahedral tilting, however, successfully realizes an ultra-stable perovskite film upon treatment.

To further assess the microscopic interaction between DMPESI and the FAPbI_3_ film, we conducted solid-state magic angle spinning (MAS) NMR measurements (^1^H NMR and ^13^C NMR)^31^. High-resolution ^1^H MAS NMR spectra of thin films of FAPbI_3_ passivated with DMPESI are shown in Fig. 2f–h. Whereas the aromatic signals of DMPESI (6–8.5 ppm) overlap with those of FA in Fig. 2f, the spectrum (0–5 ppm) contains signals originating from the aliphatic part of DMPESI. There is a qualitative difference in the aliphatic region between DMPESI and DMPESI-treated FAPbI_3_ film, indicating that in the latter case, DMPESI is in a different chemical form. Moreover, we conducted a ^1^H–^1^H spin-diffusion experiment that probes proximities via magnetic dipole–dipole interactions between protons (Fig. 2g). Despite the substantial signal overlap mentioned above, it is clear that the aliphatic signal of DMPESI at 3.4 ppm has an intermolecular dipolar coupling to FA rather than only an intramolecular dipolar coupling to the aromatic protons of DMPESI. This is evident based on the shape of the cross-peak, which has two components whose position and intensity ratio matches exactly those of the FA signal (Fig. 2g, green trace). To further corroborate the interaction of DMPESI with FAPbI_3_, we next carried out ^13^C MAS NMR measurements on drop-cast films (Fig. 2h), which shows substantial changes to the structure of DMPESI in the solid material. As shown from the cross polarization (CP) spectrum, which detects exclusively rigid local environments, the aromatic and aliphatic DMPESI peaks are shifted and broadened relative to DMPESI, revealing the interaction and/or reaction between DMPESI and FAPbI_3_. More detailed discussions of the solid-state NMR data are provided in Supplementary Note 3.

Furthermore, to investigate the composition of the new species formed upon DMPESI treatment, we synthesized various stoichiometries of the possible low-dimensional perovskites and compared their XRD patterns, as indicated in Supplementary Fig. 14. Interestingly, the two broad peaks (4.3° and 5.5°) observed in the perovskite film treated with a more than optimal amount of DMPESI (10 mg ml^−1^) cannot be assigned to any single low-dimensional perovskite species but appear at the same diffraction angles in a 1:5 mol/mol mixture of DMPESI and FAPbI_3_. This result indicates that unlike ammonium salts, which commonly form pure low-dimensional perovskites upon surface treatment, DMEPSI is prone to reacting with FAPbI_3_ to form a mixture species, which can be further evidenced by the very similar surface morphology (Supplementary Fig. 15), compared with the 10 mg ml^−1^ DMPESI-treated perovskite film as indicated in Supplementary Fig. 2. This is also in agreement with DFT calculations, which reveals the propensity of DMPESI to establish intermolecular interactions at high coverage in comparison with other ammonium salts (that is, PEAI) that tend to form two-dimensional phases at the surface of the 3D perovskite.

## Optoelectronic properties of perovskite films and devices

**Fig. 3:**
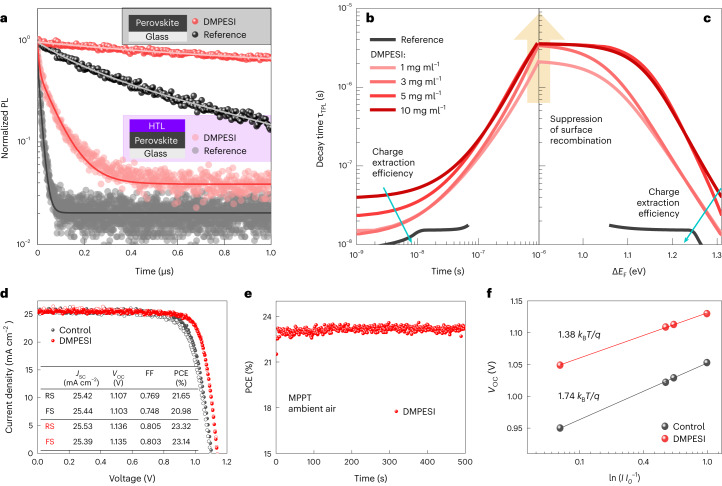
Optoelectronic properties of perovskite films and photovoltaic devices performance. **a**, Transient PL decays of reference and DMPESI-passivated (3 mg ml^−1^) perovskite films on glass with and without HTL. The fits are obtained from the multi-exponential decay function, which were used to calculate the differential lifetimes. The saturated coloured balls correspond to PL decays of perovskite on glass (shown in grey box) with (red) and without (black) DMPESI. The non-saturated coloured circles correspond to PL decays of perovskite on glass with an HTL on top (shown in lilac box). **b**,**c**, Differential decay time of passivated perovskite films with HTL plotted as a function of time after excitation (**b**) and quasi-Fermi level splitting (**c**). As the concentration of the DMPESI increases, the suppression of the surface recombination increases as well (up to 3 mg ml^−1^), but the charge extraction efficiency decreases. **d**, *J*–*V* curves of the control (black) and (3 mg ml^−1^) DMPESI-treated (red) devices, reverse scan (RS) and forward scan (FS) are indicated as solid symbols and open symbols, respectively. Inserted table summarized the corresponding photovoltaic parameters. **e**, PCE of the device employing DMPESI (3 mg ml^−1^) treatment device at maximum power point as a function of time at room temperature (r.t.), under ambient condition, without encapsulation. **f**, *V*_OC_ versus logarithm of light intensity of the solar cells with and without DMPESI treatment with corresponding linear fits, from which the slopes determined by the ideality factors (1.38 for DMPESI and 1.74 for control) were found. *k*_*B*_ is the Boltzmann constant, T is the temperature and *q* is the elementary charge.

To investigate the optoelectronic properties of the DMPESI-treated perovskite films with and without hole-transporting layer (HTL), time-resolved PL-decay measurements have been carried out, as shown in Fig. 3a and Supplementary Fig. 16, respectively. Evidently, moderate quantities of DMPESI result in effective prolongation of charge-carrier lifetime under high-injection regime, indicating high quality of the perovskite films with notably reduced non-radiative recombination. To disentangle between different competing processes leading to excess charge-carrier depopulation, we utilized a differential lifetime (*τ*_TPL_) approach pioneered by Kirchartz et al^32,33^. Because differential lifetime highly depends on the charge-carrier injection and PL derivative over time, it provides additional information about the charge-carrier dynamics on different timescales, such as second-order radiative and first-order trap-assisted recombination mechanisms. The synergy of differential lifetime analysis with a numerical simulation (description of both methods can be found in Supplementary Note 4) reveals that DMPESI-treated samples have >16 times less surface recombination. Because the introduction of charge-selective layers should not cause any additional non-radiative recombination and, hence, quench the PL intensity at longer timescales under open-circuit condition^34^. We attribute the prolongation of *τ*_TPL_ by DMPESI treatment shown in Fig. 3b to its effective suppression of surface states at the perovskite/HTL interface. However, thicker DMPESI layers (>3 mg ml^−1^) hinder the charge extraction efficiency (visible at timescales <10^−8^ s), due to charge accumulation at perovskite/DMPESI contact (Supplementary Fig. 18) without further reduction in interfacial recombination, as seen by the convergence of differential lifetimes of samples with various DMPESI concentrations at ${\tau }_{\mathrm{TPL}}\approx 3.5\,\mu\mathrm{ s}$ (Fig. 3c). Therefore, the layer produced by 3 mg ml^−1^ DMPESI solution strongly suppresses the non-radiative recombination at perovskite/HTL interface and, simultaneously, allows an efficient hole extraction by an HTL.

PSC devices were fabricated with DMPESI treatment (under various conditions) by employing the n–i–p architecture of fluorine doped tin oxide (FTO)/cp-TiO_2_/mp-TiO_2_/3D perovskite/DMPESI/spiro-OMeTAD/Au, in comparison with the reference cells and the PEAI treated ones. Statistical box charts of the photovoltaic parameters and the corresponding champion values are shown in Supplementary Fig. 19 and Supplementary Tables 6 and 7, respectively. Compared with the control devices, the devices post treated with trace amount (1 mg ml^−1^) of DMPESI present slight improvements in open-circuit voltage (*V*_OC_) and fill factor (FF), however, a large amount (10 mg ml^−1^) of DMPESI results in inferior values in all PV parameters, which we attribute to the blocked charge transfer upon aggregation and formation of a thick interlayer between perovskite and HTL, as observed in cross-section SEM in Supplementary Fig. 20. This result is also in agreement with the time-resolved PL measurement discussed above. Both *V*_OC_ and FF are substantially increased by introducing moderate quantities, especially 3 mg ml^−1^ of DMPESI, leading to the best reverse PCE of 23.32% and forward PCE of 23.14%, as summarized in the table in Fig. 3d. The PCE is stabilized at 23.3% under maximum power point, as shown in Fig. 3e. Incident photon-to-current conversion efficiency was measured and shown in Supplementary Fig. 21, which demonstrates efficient light harvesting across the absorption spectrum and has an integrated value agreeing with the measured *J*_SC_ from the *I*–*V* measurement. It is worth noticing that the hysteresis of the treated devices becomes negligible in comparison with the control devices. In addition, Fig. 3f shows a linear relationship between *V*_OC_ and the logarithm of light intensity for both the control and treated PSCs. From this measurement, the diode ideality factor (*n*_id_) of the control cell was found to be 1.74, whereas the treated cell has a *n*_id_ of 1.38. Such reduction in *n*_id_ suggests an effective suppression of the trap-assisted recombination, which affects mainly *V*_OC_ and FF losses^35,36^. On the basis of the reconstructed pseudo-*J*–*V* curves, as shown in Supplementary Fig. 22, the FF losses associated with charge transport remain nearly unchanged in the presence of DMPESI, whereas the non-radiative recombination FF losses are reduced by a factor of two. We attribute the suppression of non-radiative recombination to a strong reduction in trap density, which has been confirmed by the space-charge-limited current measurements (Supplementary Fig. 23) and DFT calculations (Supplementary Fig. 11), agreeing well with the reduction in energy tail states and Urbach energy (Supplementary Fig. 24). Combining with the analysis shown earlier, we postulate that the introduction of DMPESI on the perovskite surfaces effectively passivates the disordered states at the interfaces and thus strongly reduces non-radiative recombination to improve device *V*_OC_ and FF.

## Durability of the PSCs

**Fig. 4:**
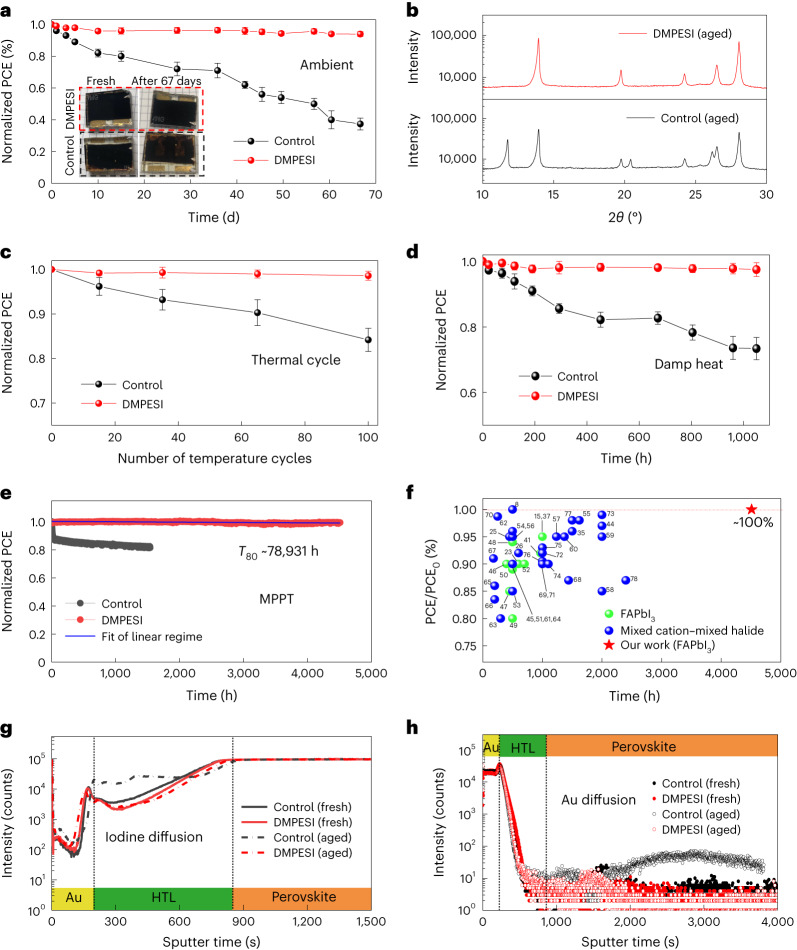
Durability of the PSCs. **a**, Dark shelf stability of unencapsulated control and DMPESI-treated PSCs and inserted photos are the devices before and after ageing in ambient condition at r.t. with R.H. around 20–40%, five devices for each condition, the initial device PCEs are 23.02 ± 0.26% (DMPESI treated) and 21.03 ± 0.48% (control). **b**, XRD patterns of shelf-aged control and DMPESI-treated PSCs. **c**, Temperature cycling (25–85 °C) test of encapsulated control and treated devices, five devices for each condition. **d**, Damp heat test (85 °C, 85% R.H.) of the encapsulated control and treated devices, five devices for each condition. The initial PCEs of temperature cycling and damp heat tested devices are 22.24 ± 0.22% (DMPESI treated) and 20.12 ± 0.42% (control). Data are presented as mean values ± SEM for **a**, **c** and **d**. **e**, Long-term operational stability of the unencapsulated control and treated devices under MPPT with continuous 1-sun illumination under N_2_ flow at room temperature. The linear fitting of DMPESI-treated device maximum power point stability, the initial device PCE is 22.68% (DMPESI treated) and 20.43% (control). **f**, Operational stability of state-of-the-art highly efficient (PCE > 22%) PSCs extracted from literature^8,15,23,25,26,35,37,41,44–78^ (perovskite composition based on FAPbI_3_ marked in green dots; mixed cation–mixed halide marked in blue dots; our work marked in red star).ToF-SIMS depth profiles of the fresh and aged (control and treated) devices of **g**, I^−^ and **h**, Au. Source data

To investigate the stability of the PSCs, we first tracked their shelf-life stability by storing the unencapsulated devices in ambient air with R.H. of 40% ± 20% for more than two months. Figure 4a shows that the PCE of a control device without any post treatment decreased by ∼60% after ageing for 67 days, whereas the treated device with 3 mg ml^−1^ DMPESI treatment retains 94% of its initial PCE value. Visual inspection of the devices before and after ageing shows that the control device turned light brown, indicating a phase transformation resulting in lower light absorption in the visible part of the spectrum. The DMPESI-treated device remained completely black, which was confirmed by XRD (Fig. 4b). A thermal-stability test was also performed by heating the unencapsulated PSCs at 60 °C with R.H. of less than 30%. From the results in Supplementary Fig. 25a, the treated cell retained more than 96% of its initial PCE, whereas the control cell retained less than 85% after 400 h of ageing. Moreover, we replaced the hole-transporting material (spiro-OMeTAD) with the more thermally stable material of poly(bis(4-phenyl)(2,4,6-trimethylphenyl)amine) (PTAA) for harsh thermal-stability tests. As shown in Fig. 4c, we observed a dramatic stability improvement in the DMPESI-treated devices (encapsulated) under thermal cycling (between 25 °C to 85 °C, procedure shown in Supplementary Fig. 25b), following the ISOS-T-1 stability protocol^7^. Furthermore, the encapsulated treated device also successfully passed the damp heat test (85 °C at 85% R.H. condition) for over 1,000 h with less than 5% loss in PCE, as shown in Fig. 4d.

To further examine the treated FAPbI_3_-based PSCs under intensive light soaking and gain insights into the intrinsic degradation mechanisms, we exposed the cells to maximum power point tracking (MPPT) under continuous 1-sun illumination in an inert atmosphere with N_2_ flow at room temperature. As presented in Fig. 4e and the corresponding current-voltage (*J*–*V*) parameters in Supplementary Fig. 26, an initial rapid burn-in regime within the first 30 h is clearly observed in the control cell, leading to a PCE loss of 12% of its initial value, which is similar to other reports on FAPbI_3_ PSCs in literature^7,37^. This is typically attributed to the redistribution of charged defects^38^. Subsequently, a slower decay is present in the control cell, owing to irreversible chemical reactions through migration, which gradually cause irreversible degradation of the device, leading to a 20% loss after operational ageing for 1,500 h. In stark contrast, the treated device shows an extraordinary long-term operational stability under the same ageing condition, with negligible PCE drop (less than 1%) after half-year MPPT ageing for over 4,500 h. This result makes our device among the most stable, high-efficiency PSC reported to date, as illustrated in Fig. 4f (more details summarized in Supplementary Table 8). A similar result is also obtained by measuring other cells within the same batch and different batches, as shown in Supplementary Fig. 27. Making a linear extrapolation of these data yields a theoretical *T*_80_ of over nine years (Fig. 4e) under continuous 1-sun illumination, which would correspond to a photon flux of an outdoor PV installation in Sweden or Germany (1,000 kWh m^−2^ per year) of over 78 years.

On the basis of the combination of results from SED, transient PL and PL-decay analysis, we attribute the initial burn-in effect present in the reference samples to lower energy barrier for I^−^ migration and their accumulation of iodide interstitials (I_i_^−^ and I_i_^+^) at the surfaces. As recently demonstrated, the phase impurities, often found at the grain boundaries, seed the degradation under illumination by trapping the photoexcited charge carriers and forming I_2_(refs.^39–41^). DMPESI post treatment effectively inhibits this mechanism by passivating the surface states and sequestrating mobile I^−^ ions at the grain boundaries, preventing an accumulation of interstitial iodide at the phase impurities and thus circumventing degradation^42^. Consequently, this leads to reduced rate of non-radiative recombination processes in perovskite, which also remain constant even after long time duration (Fig. 1k), resulting in a stable PSC performance without the burn-in effect (Fig. 4e).

Furthermore, undesirable migration, especially of iodide ions from the perovskite bulk and of gold from the metal contact, has been investigated as a principal factor that causes deleterious effects on the stability of metal halide perovskites^43,44^. Therefore, time-of-flight secondary ion mass spectroscopy (ToF-SIMS) elemental depth profiles have been carried out for both devices (control and treated) before and after the continuous operational ageing process, and the full spectra are shown in Supplementary Fig. 28. It is observed that both the as-prepared control and treated cells have similar signals occurrence for I^−^ and Au. However, for the control cell after ageing, the signals of I^−^ and Au are, as expected, substantially increased in the HTL layer and perovskite layer (Fig. 4g,h), respectively, which provides direct evidence for ion migration within the layers of the device. Such severe ion diffusion creates not only deep trap states but also shunts across the cell, leading to a dramatic PCE drop, especially in *V*_OC_ and FF, as also in accordance with the photovoltaic parameters measured throughout the MPPT ageing test. However, in sharp contrast, negligible I^−^ and Au diffusion signals of diffusing species have been detected for the treated cells after ageing. This evidence clearly indicates that the introduction of DMPESI can effectively suppress the iodide ions and gold migration and improve the device stability. Moreover, surface treatment with DMPESI also shows positive effects on device performance and stability on other perovskite compositions, as shown in Supplementary Fig. 29.

## Conclusions

In conclusion, we have shown that a single surface treatment step with a sulfonium salt leads to stabilization of the black phase of FAPbI_3_. We elucidated that its mechanism of action is related to its interaction with the perovskite surface. Both the perovskite bulk through formation of an additional intragranular species at the grain boundaries and forming a robust interlayer between perovskite and HTL efficiently blocks mobile ion migration and protects the perovskite film from external atmosphere. As a result, it enables long-term stabilization of highly efficient PSCs, especially under moisture and light-soaking conditions, which also provides a strong indication that perovskite films exhibit the potential to fulfil industrial protocols and compete with Si-based solar cells for further large-scale and industrial production. Moreover, the in-depth understanding of such unexplored sulfonium-based molecules opens up more possibilities in material science and engineering, which is important and encouraging in the field, towards further stabilization of future perovskite-based optoelectronic devices.

## Methods

### Synthesis of DMPESI

2-Mercaptophenylenthane (2.0 g, 14.5 mmol, TCI), NaOH (1.0 g, 25.0 mmol, Alfa) tetrabutylammonium iodide (200 mg, 0.5 mmol, Sigma-Aldrich) were added into the mixed solution of toluene (10 ml, Sigma-Aldrich) and H_2_O (10 ml). Iodomethane (2.26 g, 16.0 mmol, Acros) was added dropwise into the mixture. After stirring for 12 h at room temperature, brine (50 ml) was added into the solution and the mixture was extracted by Et_2_O (30 ml × 3, Acros). After the combined organic phase was dried over MgSO_4_, the solvent was removed under reduced pressure to give the product (1.93 g, 82% yield) without further purification. Colourless liquid; ^1^H NMR (400 MHz, CDCl_3_) δ 7.25–7.19 (m, 2H), 7.17–7.11 (m, 3H), 2.87–2.78 (m, 2H), 2.68 (t, *J* = 7.9 Hz, 2H), 2.05 (s, 3H). Methyl(phenethyl)sulfane (1.80 g, 11.8 mmol) was dissolved in EtOH (10 ml, Acros) at room temperature, iodomethane (1.76 g, 12.0 mmol, Acros) was added dropwise into the solution. After the mixture was stirred for 24 h, Et_2_O (30 ml, Acros) was added into the solution. The solid was filtered and washed by diethyl ether three times to afford the final product. White solid; ^1^H NMR (400 MHz, DMSO) δ 7.36 (d, *J* = 4.4 Hz, 4H), 7.27 (ddd, *J* = 8.7, 5.0, 3.8 Hz, 1H), 3.72–3.59 (m, 2H), 3.10 (dd, *J* = 8.8, 7.0 Hz, 2H), 2.97 (s, 6H); ^13^C NMR (101 MHz, DMSO) δ 137.98, 129.22, 129.15, 127.58, 43.43, 29.47, 24.73.

### Synthesis of FAPbI_3_ powder

Formamidinium acetate (20.8 g, 0.20 mol, Sigma-Aldrich) was dissolved in ethanol (30 ml, Acros) at 0 °C, HI solution (57% in water, 40 ml, Sigma-Aldrich) was added dropwise into the solution. After 30 min, the solution was removed to room temperature and stirred for another 1 h. The solvent was removed by using a rotary evaporator at 60 °C; the solid was recrystallized by mixture solution of ethanol and diethyl ether (Sigma-Aldrich) three times. Then the formamidinium iodide was dried under vacuum for 2 h. FAI (2.75 g, 16 mmol) and PbI_2_ (7.37 g, 16 mmol) were dissolved in the anhydrous 2-methoxylethanol (20 ml, Sigma-Aldrich), then the solution was stirred at 140 °C for 2 h, anhydrous toluene (40 ml, Sigma-Aldrich) was added dropwise. The black FAPbI_3_ powder was filtered and dried under vacuum for 2 h, the black powder was stored in argon glovebox.

### Substrate

Fluorine doped tin oxide (FTO) substrates (NSG-10) were chemically etched by zinc powder and 4 M HCl solution and sonicated in 2% Hellmanex water solution for 30 min, acetone for 15 min and ethanol for 15 min, respectively. Then, all substrates were further cleaned by UV–ozone for 15 min. Then a compact TiO_2_ layer was deposited on cleaned FTO substrates via spray pyrolysis deposition from a precursor solution of titanium diisopropoxide bis(acetylacetonate) (Sigma-Aldrich) in anhydrous ethanol (Acros), with oxygen as carrier gas. Substrates were heated at 450 °C and kept at this temperature for 15 min before and 30 min after the spray of the precursor solution then left to cool down to room temperature. Mesoporous TiO_2_ layer was spin coated at 3,000 r.p.m. for 30 s, with the acceleration rate of 1,500 r.p.m. s^−1^, using a 30 nm TiO_2_ paste (Dyesol 30 NR-D) diluted in ethanol with 1:6 volume ratio. After the spin coating, the substrates were dried at 80 °C for 10 min and then sintered at 450 °C for 30 min under dry air flow.

### Perovskite layer

The perovskite precursor solution was prepared by dissolving FAPbI_3_ powder (1.8 M) and MACl (0.63 M, Dyenamo) into mixed solvent of DMF and DMSO (DMF:DMSO = 4:1 v/v, Acros). The perovskite solution was spin coated at 6,000 r.p.m. for 50 s with pouring diethyl ether (1 ml, Acros) as an anti-solvent at 15 s of spin-coat process. Then the substrates were annealed at 150 °C for 10 min in dry air. The DMPESI was dissolved in chloroform (Acros) with different concentration and the solution was spin coated at 4,000 r.p.m. for 20 s on the as-prepared perovskite films and dried on a hot plate at 100 °C for 10 min.

### Hole-transporting layer and Au top contact

The substrates were cooled down to room temperature after annealing the perovskite. The hole-transporting solution was deposited by spin coating at 4,000 r.p.m. for 20 s as hole-transporting material. Spiro-MeOTAD: 90 mg spiro-MeOTAD (Xi’an Polymer Light Technology) was dissolved in 1 ml chlorobenzene, doped by 20.6 μl bis(trifluoromethylsulfonyl)imide lithium salt solution (520 mg ml^−1^ LiTFSI in acetonitrile, Sigma-Aldrich) and 35.5 μl 4-tert-butylpyridine (tBP, Sigma-Aldrich). PTAA: 10 mg PTAA (Xi’an Polymer Light Technology) was dissolved in 1 ml, doped by 1.6 μl bis(trifluoromethylsulfonyl)imide lithium salt solution (520 mg ml^−1^ LiTFSI in acetonitrile, Sigma-Aldrich) and 2 μl 4-tert-butylpyridine. Finally, 80 nm of Au top electrode was deposited through thermal evaporator under high vacuum with an active area of 0.16 cm^2^.

### PSC characterization

The solar cell devices were measured using a 300 W Xenon light source (Oriel). The spectral mismatch between AM 1.5 G and the solar simulator was calibrated by a Schott K113 Tempax filter (Prazosopms Gas and Optik GmbH). The light intensity was calibrated with a silicon photodiode with an infrared-cut-off filter (KG2, Schott). Current–voltage characteristics were applied by an external voltage bias while measuring the corresponding current with Keithley 2400 under ambient air condition. The voltage scan rate was 50 mV s^−1^. The devices were covered with a black metal mask with an active area of 0.16 cm^2^. Incident photon-to-current efficiency was carried by a commercial apparatus (Aekeo-Ariadne, Cicci Research s.r.l.). The top-view and cross-section morphologies of the samples were characterized using a high-resolution scanning electron microscope (Zeiss Merlin) with an in-lens secondary electron detector.

### XRD and GIWAXS measurement

The X-ray diffraction patterns were recorded with PANalytical Empyrean system with a PIXcel-1D detector, Bragg–Brentano beam optics and parallel beam optics. Light source is from copper Kα beam filtered with nickel β filter. Diffraction spectra were characterized between 2θ of 10° and 70° at a scan rate of 1° per minute with the step width of 0.02°. GIWAXS were measured on a Bruker D8 Discover Plus instrument equipped with a rotating anode and a Dectris Eiger2 500 K detector. The primary beam path was collimated by a 1.0 mm micromask (after a focussing Göbel mirror), followed by a 0.5 mm micromask, followed by a 0.3 mm double-pinhole collimator. The detector was placed at 178 mm from the sample and 2D images were acquired for 300 seconds at 2° theta (incidence angle). Two-dimensional images were integrated using EVA software. Photoelectron spectroscopy measurements were performed in a custom-built ultra-high vacuum system. The pass energy was 10 eV, and the samples were grounded during the measurement.

### SED measurement

Scanning electron diffraction (SED) data were acquired on the JEOL ARM300CF E02 instrument at ePSIC, Diamond Light Source with a Merlin/Medipix pixelated STEM detector. Utilizing the following experimental parameters: accelerating voltage = 200 kV; nanobeam alignment ( ∼ 1 mrad convergence angle); electron probe ∼5 nm; probe current ∼3.1 pA; scan dwell time 1 ms; camera length 15 cm, we achieved an electron dose per scan of ∼10 eÅ^−^^2^ at 150,000 × magnification when approximating the beam shape as a circle with a diameter of ∼5 nm. This accumulated dose is almost an order of magnitude lower than the lowest reported threshold at which the crystal structure of FAPbI_3_ begins to change (66 eÅ^−^^2^). SED data were calibrated and corrected for elliptical distortions using reference data acquired on an Au cross grating. In SED, under our experimental conditions, 1 pixel on the Merlin/Medipix pixelated STEM detector is ∼0.0059 Å^−^^1^. SED diffraction data were analysed in pyXem. In the manuscript, we refer to annular dark-field (ADF) images in reference to SED data presented in Fig. 2. ADFs are reconstructed from SED data by plotting the integrated diffracted intensity as a function of probe position, excluding the directly transmitted beam. The samples were prepared on SiN windows from Norcada (part number: NT025X) with 0.65 M perovskite precursor; the spin-coating process is same with perovskite film fabrication above. SED integrates through the thickness of the film and thus does not provide any depth resolution in its measurements.

### Hyperspectral photoluminescence map

Wide-field, hyperspectral photoluminescence microscopy was performed using a Photon etc. IMA system. The sample was excited with a 405 nm continuous wave laser shaped to have a top hat profile larger than the field of view of the camera so the area of interest was uniformly illuminated. The excitation was focused onto the sample using an Olympus 100× air objective (MPLFLN100×) to produce an illumination intensity of 67 mW cm^−^^2^, equivalent to approximately 1-sun illumination for a material with the bandgap of FAPbI_3_. A volume Bragg grating was used to spectrally split the light at each point on the sample. First a hyperspectral scan of a sample was performed, then the sample was continuously illuminated for a period of time and broadband luminescence images were intermittently captured every 1.5 seconds. Once this light-soaking period was complete, a final hyperspectral scan was once again performed.

### Solid-state NMR

Solid-state magic angle spinning (MAS) NMR spectra of ^1^H (1,000.4 MHz) were recorded on a Bruker Avance Neo 23.5 T spectrometer equipped with a 1.3 mm MAS probe using 100 kHz RF field amplitude. For the ^1^H–^1^H spin-diffusion measurement, a recycle delay of 2 s, mixing delay of 2 s, 150 slices and 16 scans per slice were used. The 2D dataset was processed with 100 Hz of Lorentzian apodization in the directly detected dimension. ^1^H chemical shifts were referenced to the ^13^C chemical shift of solid adamantane (38.48 ppm for the CH_2_ signal) using the ratio of the gyromagnetic ratios in accordance with the International Union of Pure and Applied Chemistry (IUPAC) recommendation. ^13^C echo and ^1^H–^13^C CP MAS spectra were recorded on a Bruker Avance Neo 14.1 T (150.7 MHz) spectrometer equipped with a 4 mm CP MAS probe and referenced to solid adamantane. Seventy-four kHz ^1^H decoupling was used. The recycle delays and numbers of scans are given in Supplementary Table 2. The rotors were spun using dry nitrogen. Two types of sample were used for NMR: (1) spin-coated passivated thin films prepared according to the same procedure as in device fabrication (^1^H measurements) and (2) drop-cast films (^13^C measurements, to obtain a larger amount of material).

### Stability test

The operational stability of the devices was measured under a white light-emitting diode lamp with biologic MPG2 potentiostat under N_2_ gas flow at maximum power point tracking (MPPT). The encapsulation of PSCs devices is using EVA as the encapsulant and using glass as the cover. For the temperature cycling test, a hot plate is using to control the temperature (R.T.–85 °C).

### Other measurements

The photoluminescence lifetime was measured via time-correlated single photon counting using a LifeSpec II (Edinburgh Instruments) fluorescence spectrometer with a picosecond pulsed diode laser (EPL-510, Edinburgh Instruments) at 510 nm wavelength. ^1^H NMR and ^13^C NMR measurements were performed on Bruker AvanceIII-400 MHz NMR spectrometer. Atomic force microscopy (AFM) measurements were carried out using an AFM (Bruker Dimension Icon) in air with controlled humidity at around 20% R.H.

### Reporting summary

Further information on research design is available in the Nature Portfolio Reporting Summary linked to this article.

### Supplementary information

Supplementary Information Supplementary Notes 1–4, Figs. 1–29, Tables 1–8 and Refs. 1–58.

Reporting Summary

Supplementary Video 1 Hyperspectral photoluminescence mapping of reference film.

Supplementary Video 2 Hyperspectral photoluminescence mapping of DMPESI-treated film.

Supplementary Data 1 Raw DFT data for Supplementary Fig. 10.

Supplementary Data 2 Geometry of FAI, DMPESI and PEAI.

Supplementary Data 3 The individual data behind the average values in Supplementary Table 7.

Supplementary Data 4 The individual data behind the average values in Supplementary Fig. 25.

Supplementary Data 5 The individual data behind the average values in Supplementary Fig. 29.

### Source data

Source Data Table 1 The individual data behind the average values in Fig. 4a,c,d.

## Data Availability

All data generated or analysed during this study are included in the published article and its Supplementary Information files. Source data are provided with this paper.
