# Supplementary material for: Multifunctional sulfonium-based treatment for perovskite solar cells with less than 1% efficiency loss over 4,500-h operational stability tests
Source: Nat Energy. 2024 Jan 4;9(2):172–83. doi: 10.1038/s41560-023-01421-6 (PMC10896729; doi:10.1038/s41560-023-01421-6)
Supplement: Supplementary file 1 — Supplementary Notes 1–4, Figs. 1–29, Tables 1–8 and Refs. 1–58. [file 41560_2023_1421_MOESM1_ESM.pdf]

nature energy

Article https://doi.org/10.1038/s41560-023-01421-6

Multifunctional sulfonium-based treatment for perovskite solar cells with less than 1% efficiency loss over 4,500-h operational stability tests

In the format provided by the authors and unedited

Table of Contents

Supplementary Note 1-4

Supplementary Figure 1-29

Supplementary Table 1-8

Supplementary References 1-58

Supplementary Note 1

The Mulliken atomic charges analysis was carried out on the isolated $DMPSE^+$ and PEA^+ cations using Gaussian09[1] with the B3LYP functional[2] along with the lanl2dz[3] basis set. Considering Mulliken charges with hydrogens summed into heavy atoms, we calculate the N charge of +0.6 e while for S is found to be +0.5 e. This is clearly confirmed by observing the electrostatic potential isodensity plot reported in Supplementary Fig. 1, where a higher charge localization is found for PEA^+ in the $-NH_3^+$ region while for $DMPES^+$ cation that is showing a more delocalized charge.

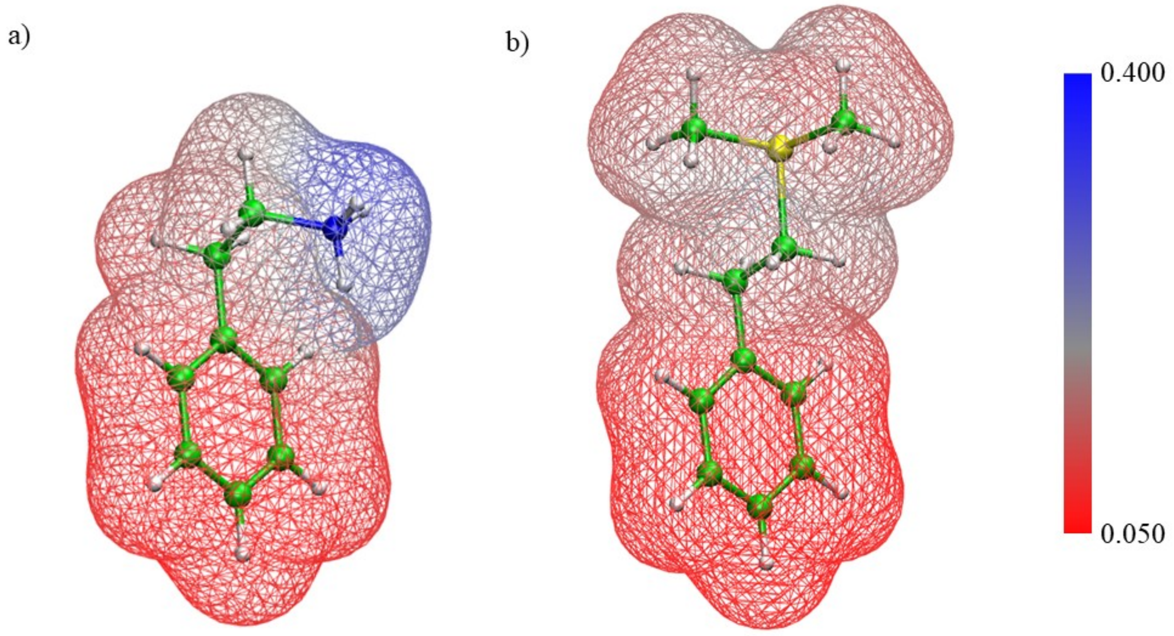

Supplementary Figure 1. Electrostatic potential isodensity plot. Isodensity plot of the electrostatic potential for (a) PEA^+ and (b) $DMPES^+$ calculated in water. The blue region indicates the positive charge localization.

To evaluate the hydrophobicity of the different cations we also estimated with Gaussian09, the tendency of binding one water molecules by simulating the interacting complex A^+ --- H_2O and calculating ΔE_{H2O} as follows:

$\Delta E_{H2O} = E(A^+ \cdot H_2O) - E(A^+) - E(H_2O)$ accordingly to the equation (2).

$$A^+ + H_2O \rightarrow A^+ \cdot H_2O \qquad (2)$$

Finally, we also evaluated the solvation energy to further confirm the hydrophobicity of the different cations, see Supplementary Table 1. ΔG_{solv} is calculated as the energy different between the total free energy in solution in water solution and the single point energy in vacuo on the geometry optimized in solvent.

Supplementary Table 1. Interaction energy with a water molecule (ΔE_{H2O}, eV), see equation (3), and solvation free energy (ΔG_{solv}, eV) for PEA^+ and $DMPES^+$.

A	ΔE_{H2O}	ΔG_{solv}
PEA^+	-0.53	-2.56
$DMPES^+$	-0.17	-2.18

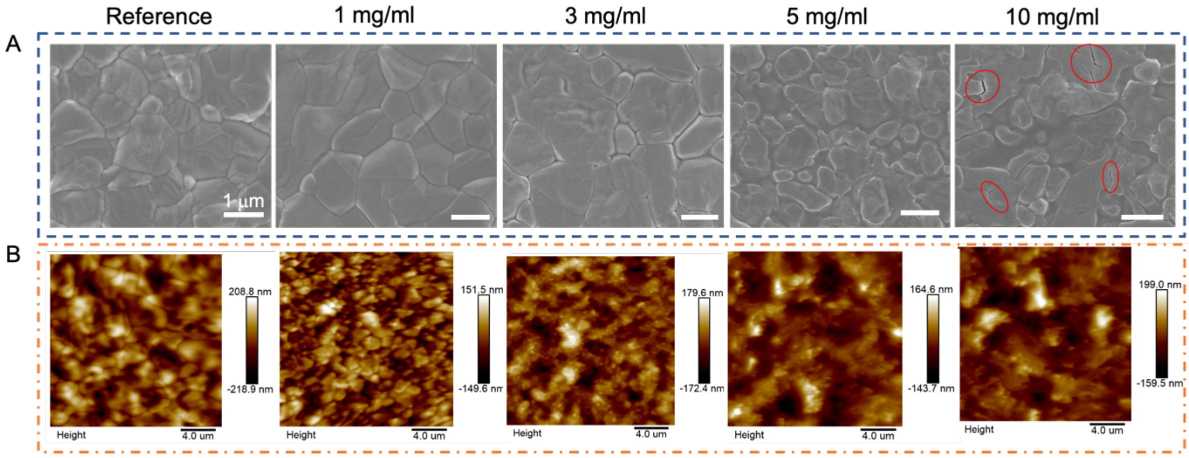

Supplementary Figure 2. **SEM and AFM images of perovskite films.** (a) Top-view SEM images of pristine perovskite film, perovskite films treated with 1 mg/mL, 3 mg/mL, 5 mg/mL, 10 mg/mL DMPESI, respectively; (b) AFM images of pristine perovskite film, perovskite films treated with 1 mg/mL, 3 mg/mL, 5 mg/mL, 10 mg/mL DMPESI, respectively.

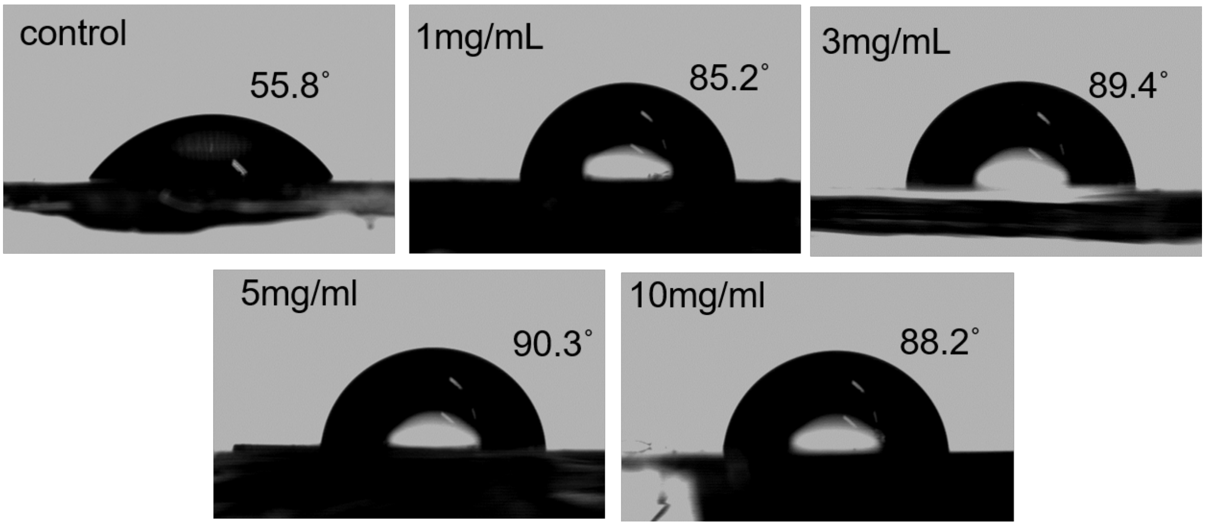

Supplementary Figure 3. **Contact angle measurements.** Contact angle of water droplet on perovskite film without and with DMPESI treatment.

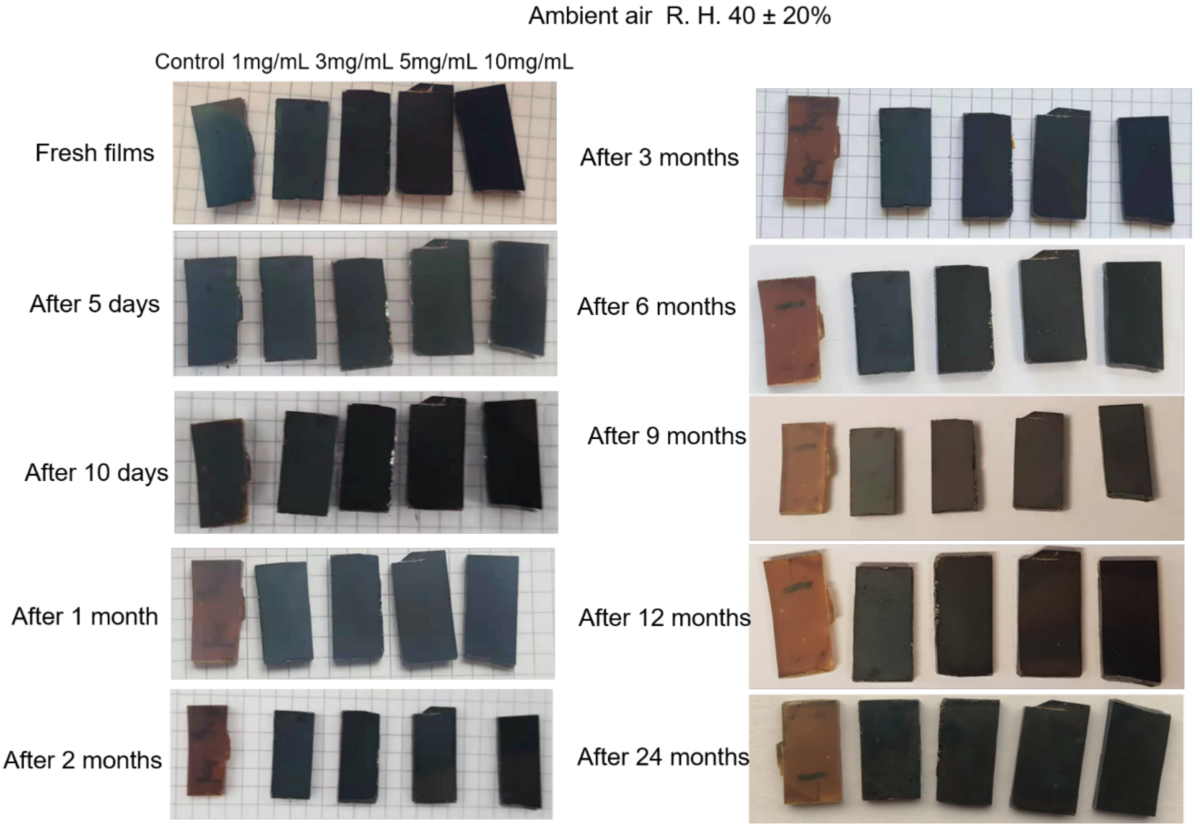

Supplementary Figure 4. Moisture stability of perovskite films under ambient condition. Photographs of unencapsulated perovskite film without and with DMPESI treatment of different concentrations (1mg/mL, 3mg/mL, 5mg/mL, 10 mg/mL) in ambient air.

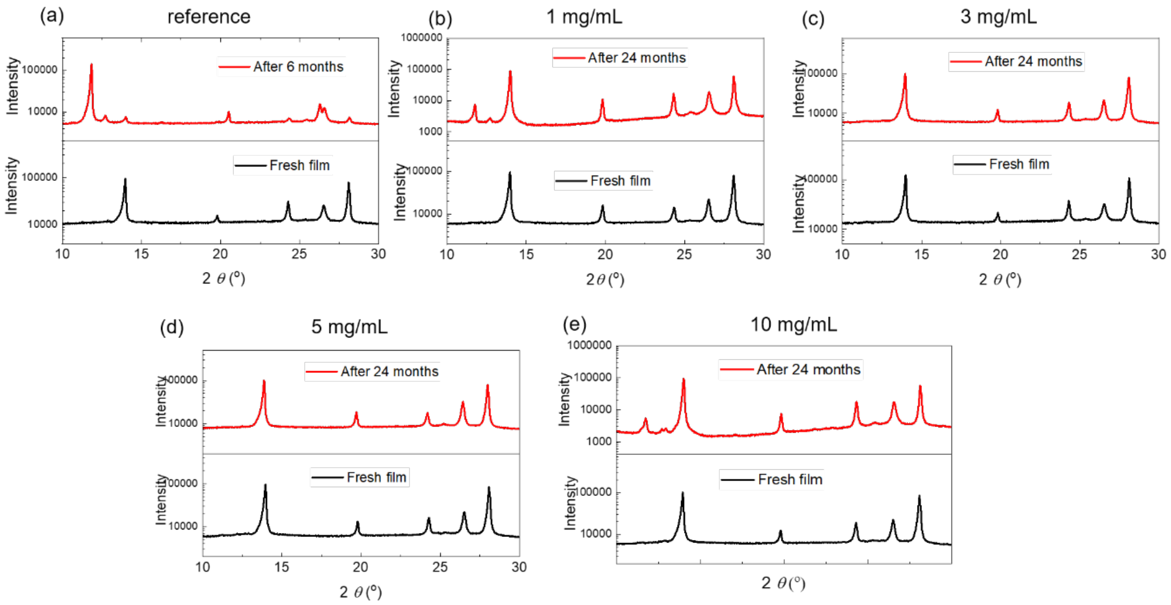

Supplementary Figure 5. **XRD patterns of perovskite films under ambient condition.** XRD patterns of (a) unencapsulated reference perovskite film and perovskite film treated with (b) 1 mg/mL, (c) 3 mg/mL, (d) 5 mg/mL, (e) 10 mg/mL DMPESI after 24-month-storage in ambient air, respectively.

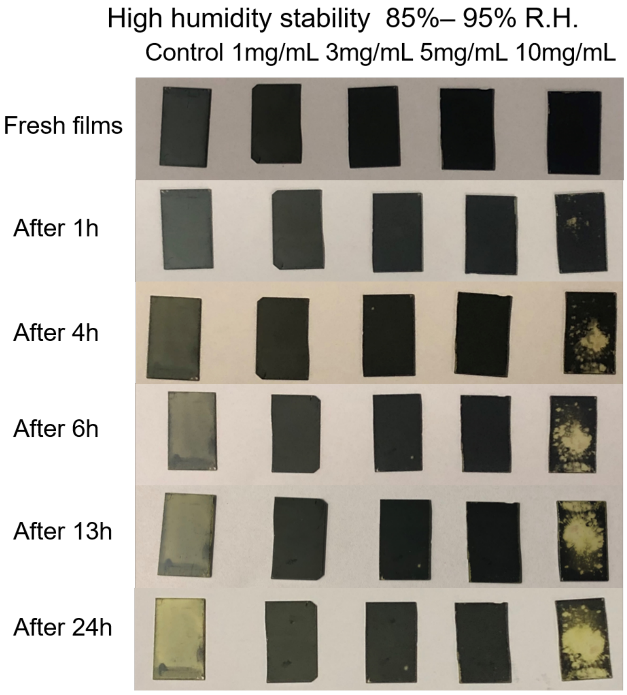

Supplementary Figure 6. **Moisture stability of perovskite films under high R.H.** Photographs of unencapsulated perovskite film without and with DMPESI treatment of different concentrations (1mg/mL, 3mg/mL, 5mg/mL, 10 mg/mL) exposing to R.H. of 85-95%.

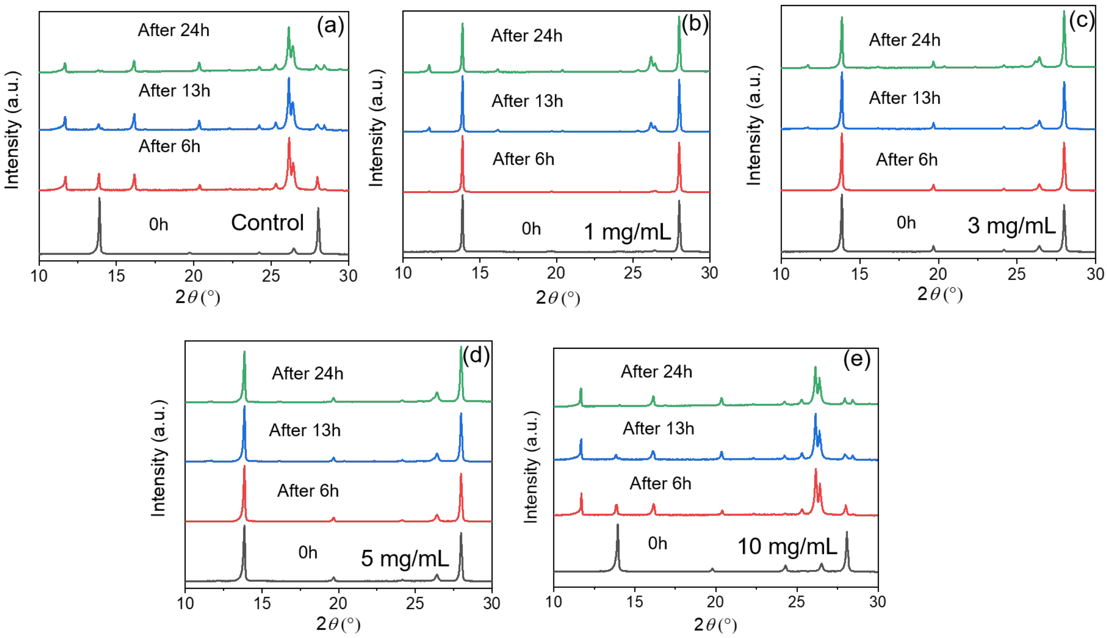

Supplementary Figure 7. **XRD patterns of perovskite films under high R.H.** XRD patterns of (a) unencapsulated reference perovskite film and perovskite film treated with (b) 1 mg/mL, (c) 3 mg/mL, (d) 5 mg/mL, (e)10 mg/mL DMPESI exposing to R.H. of 85-95%.

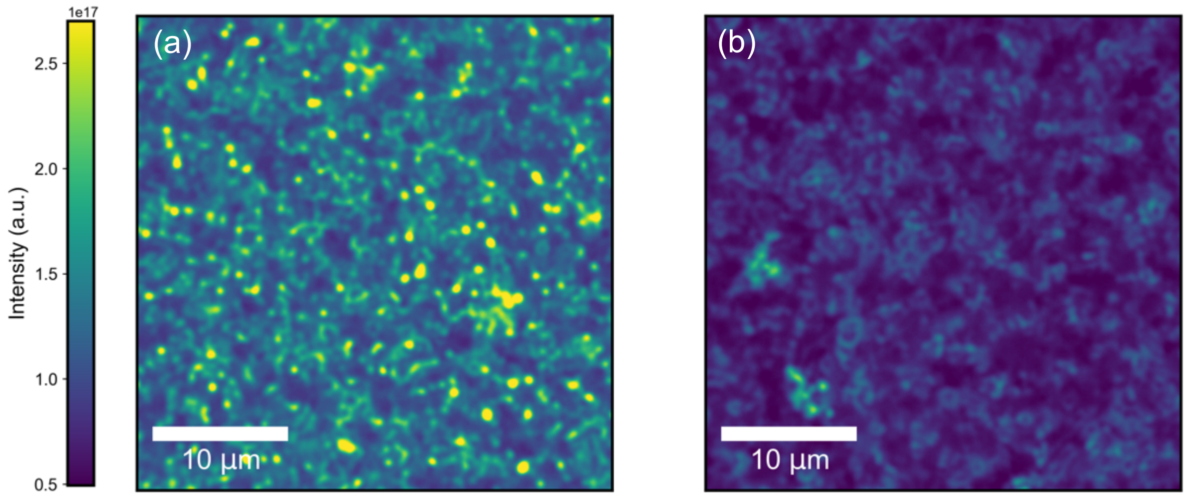

Supplementary Figure 8. **Photoluminescence maps of perovskite films.** Photoluminescence maps spectrally filtered at 1.49 eV for the (a) reference and (b) DMPESI-treated thin films showing the different morphologies between the two samples. The low energy emission appears to decorate the edges of the grains in the treated sample.

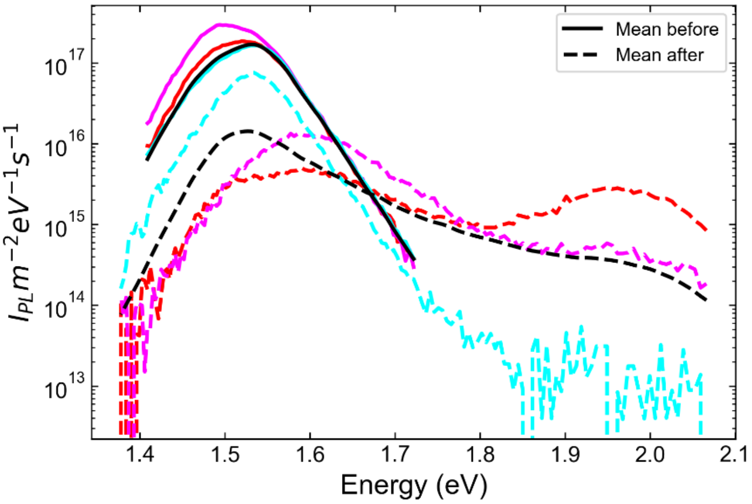

Supplementary Figure 9. **Semi-log plot of the photoluminescence intensity of perovskite films under light soaking.** Semi-log plot of the photoluminescence intensity of different regions of a reference $FAPbI_3$ before (solid lines) and after (dashed lines) ten minutes of light soaking. The cyan region retaining the highest emission simply shifts to lower intensity with no change in emission spectrum, whereas the red and magenta regions show a blue shifting to higher energy and the emergence of a new peak close to 2 eV, potentially indicative of a non-perovskite phase of $FAPbI_3$.

Supplementary Note 2

DFT calculations have been carried out on the (001) $FAPbI_3$ surface within the supercell approach by using the Perdew-Burke-Ernzerhof (PBE)[4] functional. Slabs models have been built starting from the pseudo-trigonal $FAPbI_3$ phase rotated and properly cut to reproduce a similar tetragonal phase of $MAPbI_3$, by fixing cell parameters to the experimental values[5]. This approach has been already applied previously to ensure a proper comparison with the $MAPbI_3$ systems[6]. The slab consists in 2x2 tetragonal supercell on a and b periodic directions and five layers along the perpendicular non-periodic direction where a 10 Å region of vacuum were added in all cases. Starting from the flat PbI_2- and FAI-terminated (001) surface, a full DMPESI coverage have been added.

This model and/or the following approach for determining the passivation energy were already applied in similar systems and fully tested in several papers.[7-12]

The total passivation energy (E_{tot}) is calculated following this equation:

$E_{tot} = [E_{pass\text{-}slab} - (E_{pristine} - 16^{*}E_{DMPESI})]$

where $E_{pass\text{-}slab}$ is the total energy of the DMPESI -passivated slab; $E_{pristine}$ is the total energy of the pristine PbI_2- or FAI-terminated bare surface and E_{DMPESI} is the energy of the isolate salt ion-pair. We normalize E_{tot} dividing by the number of the molecules (16) which correspond to the number of the undercoordinated Pb atoms on the two surfaces. This is the number at 100% of coverage with 8 molecules on both slab sides. We also simulate a 50% of coverage (8 molecules, 4 on both side) and 12.5% coverage (2 molecules, 1 on both side).

The intermolecular interaction energy (E_{inter})[13] is calculated as follows:

$E_{inter} = E_{layer} - 8 \times E_{DMPESI}$

where the E_{layer} is calculated by cutting the passivation layer of DMPESI and performing, on its interacting geometry, a single point energy evaluation (E_{layer}). In this case we normalize by 8 because of is the number of DMPESI ion-pairs for one passivation layer. For the lower coverage we normalize accordingly to the molecules number.

The E_{ads} is easily calculated as a difference between E_{tot} and E_{inter}. All these values are reported in Supplementary Table 2 and related geometry structure reported in Supplementary Fig. 10.

Dispersion interactions are considered on the optimized geometries using Grimmes' D3 approach[14]. Salts ion-pair geometries have been optimized using 20 Å supercells, which are large enough to decouple long range interactions Moreover, we also performed geometry optimization using D3 on the FAI, PEAI and DMPESI at 12% coverage on PbI2-terminated surface obtaining the same trend with respect to related single point energies, see italics values in Supplementary Table 2. All calculations have been carried out at Γ point.

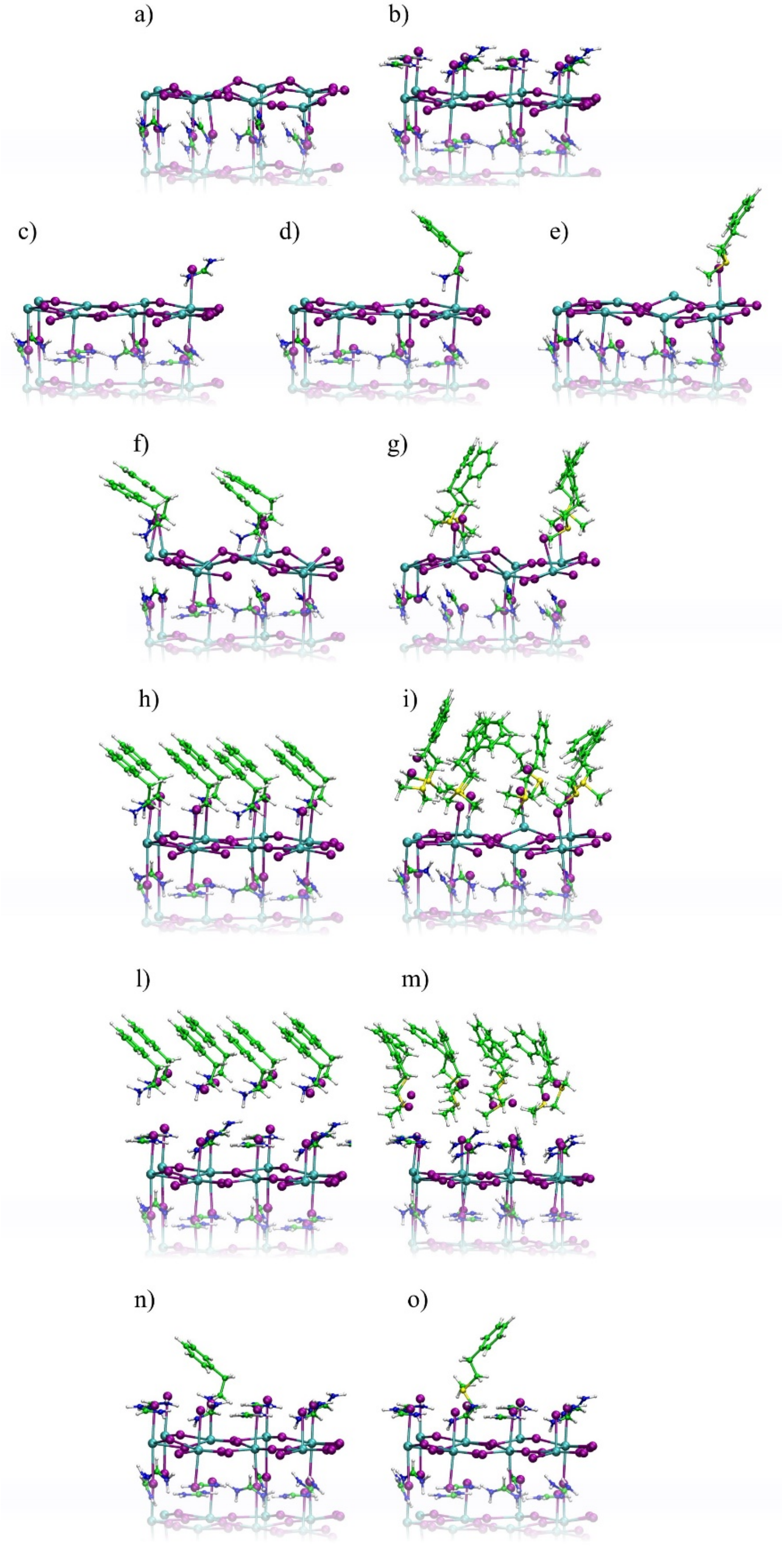
a)
b)
c)
d)
e)
f)
g)
h)
i)
l)
m)
n)
o)

Supplementary Figure 10. **Optimized geometries of $FAPbI_3$ with different passivation materials**. Optimized geometries of PbI_2-terminated (a) and the FAI-terminated (b) surface. PbI_2-terminated slab at 12.5% passivation coverage with (c) FAI, (d) PEAI and (e) DMPESI. PbI_2-terminated slab at 50% passivation coverage with (f) PEAI and (g) DMPESI. PbI_2-terminated slab at 100% passivation coverage with (h) PEAI and (i) DMPESI. FAI-terminated slab at 100% passivation coverage with (l) PEAI and (m) DMPESI. Optimized geometries of FAI-terminated surface with one vacant FAI site filled by one (n) PEAI and one (o) DMPESI. All the xyz geometries are reported in the separated raw data referring to this figure labeling. We employed experimental cell parameters[5] to build the 2x2x5 slab (a=b=17.698 Å) and the c dimension is set by adding 10Å of vacuum.

Supplementary Table 2. Passivation energy (eV) for the investigated species. Values in parentheses are calculated without D3 dispersion contribution. Values in *italics* for the 12.5% and 100% PbI2-terminated coverage are calculated including D3 dispersion also for the geometry relaxations.

Surface	Passivation molecule	E_{tot}	E_{inter}	E_{ads}
PbI_2-term. 100% coverage	FAI	1.89/*1.89* (1.47)	0.93 (0.78)	0.96 (0.59)
	PEAI	2.19/*2.22* (1.46)	1.09 (1.09)	1.10 (0.72)
	**DMPESI**	**2.21/*2.29* (1.46)**	**1.35 (0.90)**	**0.86 (0.56)**
PbI_2-term. 50% coverage	PEAI	1.71 (1.29)	0.20 (0.13)	1.51 (1.16)
	**DMPESI**	**1.93 (1.47)**	**0.59 (0.50)**	**1.34 (0.98)**
PbI_2-term. 12.5% coverage	FAI	1.61/*1.63* (1.40)	-	1.61 (1.40)
	PEAI	1.74/*1.70* (1.34)	-	1.74 (1.34)
	**DMPESI**	**2.09/*1.90* (1.70)**	**-**	**2.09 (1.70)**
FAI-term. 100% coverage	PEAI	1.28 (0.80)	1.13 (0.80)	0.15 (0.00)
	**DMPESI**	**1.69 (1.17)**	**1.48 (1.03)**	**0.21 (0.14)**
FAI-term. 1 FAI vacant*	FAI	2.34 (1.54)		
	PEAI	2.47 (1.65)		
	**DMPESI**	**2.48 (1.62)**		

* We evaluate the binding energy of one AI salt ion-pair on a FAI-terminated slab with one FAI vacancy on both sides. We adsorb the AI salt ion-pair on this vacancy site**.**

Supplementary Table 3. Salt ion-pair exchange energy (ΔE_{exc}) in eV, see equation 1 below.

AI	ΔE_{exc} w/o D3	ΔE_{exc} w D3
PEAI	-0.11	-0.12
DMPESI	-0.08	-0.13

8FAI-terminated + 1AI → 7FAI-1AI-terminated + 1FAI (1)

DFT-D3 dispersion interactions are included by performing a single point on the optimized geometries. All passivation slab models are simulated using the Quantum Espresso package[15]. PBE calculations have been performed by using ultrasoft pseudopotentials (shells explicitly included in calculations: F 2s, 2p; Br 4s, 4p; I 5s, 5p; N, C 2s, 2p; H 1s; Pb 6s, 6p, 5d) and a cutoff on the wavefunctions of 40 Ryd (320 Ryd on the charge density). Projected density of states (DOS) of the PbI_2- and FAI-terminated and passivated slabs are reported in diagrams of Supplementary Fig. 11 and have been calculated following the approach already used in our previous work[9]. The DOS in Supplementary Fig. 11 represent slab's projected DOS as a function of the energy (x axis) and of the z-coordinate orthogonal to the slab surface. The calculated DOS reported in the diagrams have been aligned to the respective VB level in all cases. All investigated structures are reported in Supplementary Fig. 10.

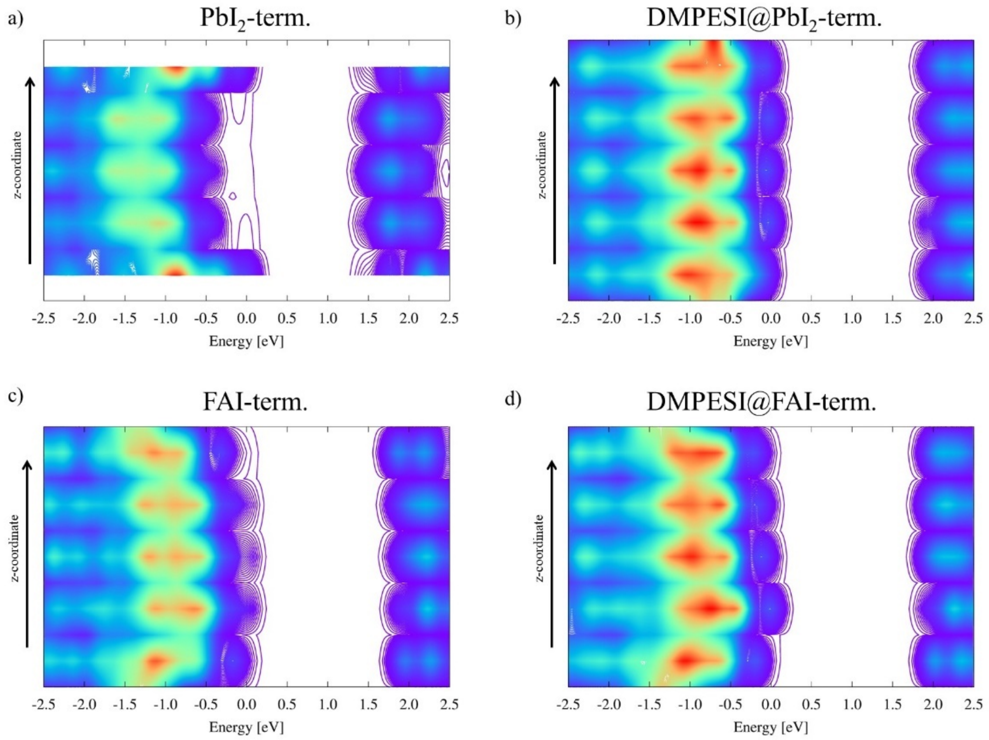

Supplementary Figure 11. Projected density of states (DOS) of the PbI_2- and FAI-terminated and passivated slabs. Isodensity contour plots of DOS layer by layer for the (a) bare PbI_2-terminated slab and the (b) relative DMPESI passivate slab; isodensity contour plots of DOS layer by layer for the (c) bare FAI-terminated and the (d) relative DMPSEI passivate slab.

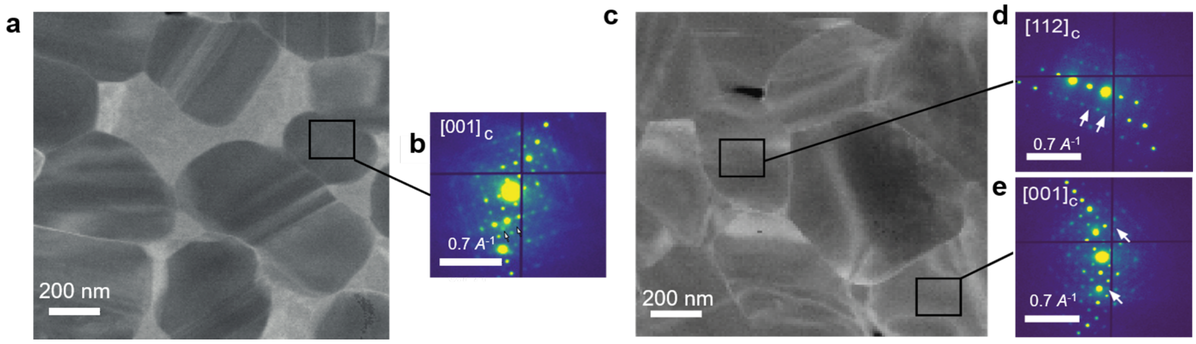

Supplementary Figure 12. SED measurement of perovskite films. **a**, Annular dark-field image reconstructed from SED data of DMPESI treated perovskite film; the ED patterns extracted from grain region are shown in **b**. **c**, Annular dark-field image reconstructed from SED data of reference film; **d** and **e** the ED patterns extracted from the grain region of a reference film.

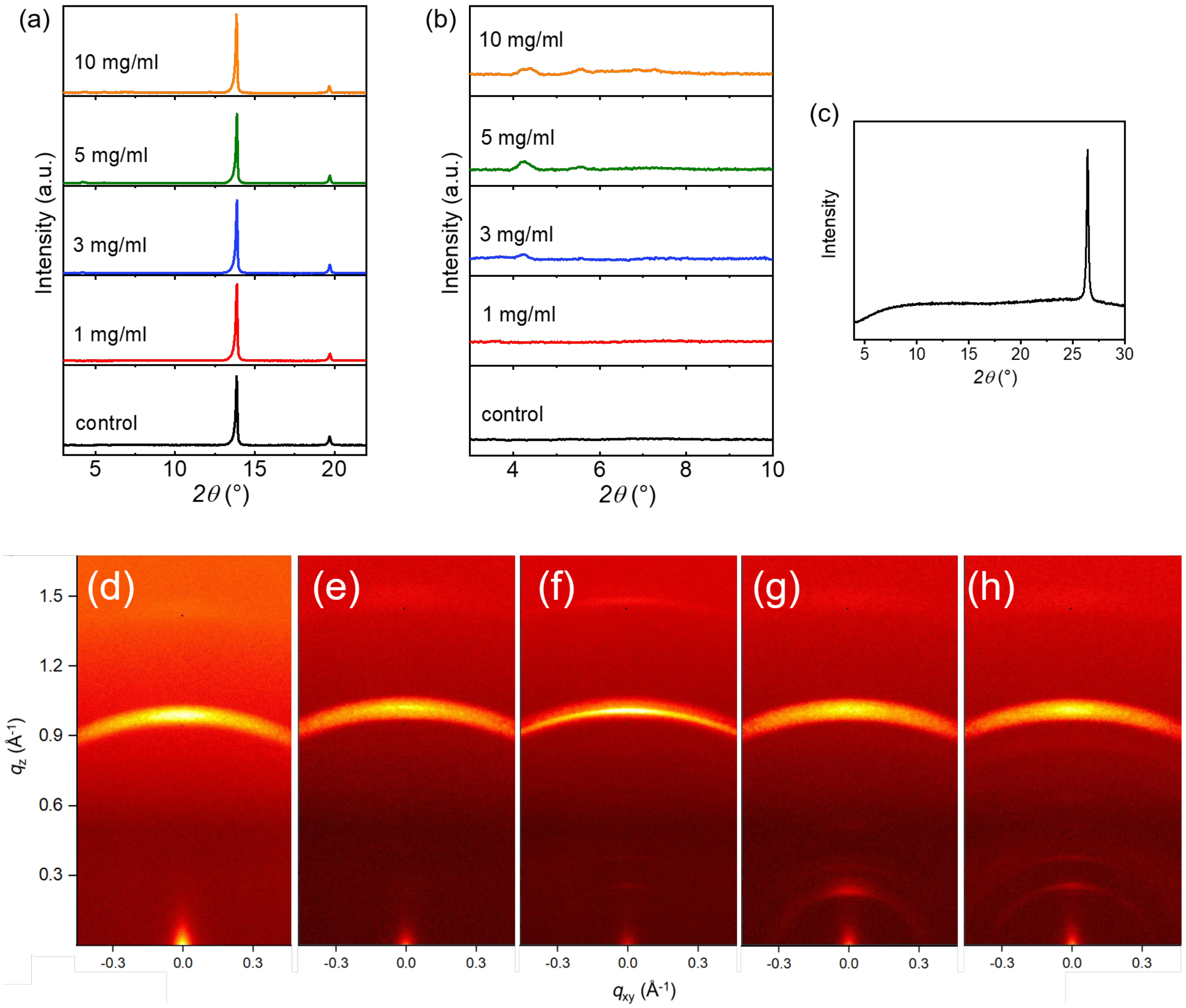

Supplementary Figure 13. **XRD patterns and GIWAXS images of perovskite films.** (a), (b) XRD patterns of reference perovskite film (black), passivated with 1 mg/mL (red), 3 mg/mL (blue), 5 mg/mL (green) and 10 mg/mL (orange) DMPESI. (c) XRD patterns of DMPESI film on the FTO glass substrate. GIWAXS images of (d) reference perovskite film and perovskite film treated with (e) 1 mg/mL, (f) 3 mg/mL, (g) 5 mg/mL, (h) 10 mg/mL DMPESI, respectively.

Supplementary Note 3

To assess the microscopic mechanism of action of DMPESI leading to the improved stability of $FAPbI_3$, we conducted solid-state Magic Angle Spinning (MAS) NMR measurements. MAS NMR is an established method for studying the atomic-level structure of dilute organic dopants in metal halide perovskites[16]. We first carried out high-resolution ^1H MAS NMR measurements on material deposited on glass slides through spin-coating using the same protocol as for the fabrication of PSCs (Fig. 2f). While the aromatic signals of DMPESI (6-8.5 ppm) overlap with those of FA, the aliphatic part of the spectrum (0-5 ppm) in principle contains only signals originating from the aliphatic part of DMPESI. There is a qualitative difference in the aliphatic region between neat DMPESI and DMPESI-doped $FAPbI_3$ thin film, indicating that in the latter case DMPESI is in a different chemical form. We also note the presence of low intensity peaks at 11.8 and 12.6 ppm, which suggest more complex chemical transformations of DMPESI and/or FA in this system. While we are unable to assign these species at present, we can assess if they are in direct atomic-level contact with the perovskite phase. To that end, we conducted a ^1H-^1H spin-diffusion (SD) experiment which probes proximities via magnetic dipole-dipole interactions between protons, which are only present if the species are present within the same phase (Fig. 2g). Off-diagonal peaks in SD spectra indicate atomic-level contact between the respective groups of protons. Despite the substantial signal overlap mentioned above, it is clear that the aliphatic signal of DMPESI at 3.4 ppm has an intermolecular dipolar coupling to FA rather than only an intramolecular dipolar coupling to the aromatic protons of DMPESI. This is evident based on the shape of the cross-peak, which has two components whose position and intensity ratio matches exactly those of the FA signal. To corroborate the reactivity of DMPESI with $FAPbI_3$, we next carried out ^{13}C MAS NMR measurements on a sample made by drop-casting to increase the amount of material (Fig. 2h). However, we note that the material obtained this way did not form a black perovskite phase and instead remained dark yellow after drop-casting process. Despite this complication, the spectrum shows substantial changes to the structure of DMPESI in the solid material. As shown in the CP spectrum, which detects exclusively rigid local environments, the aromatic and aliphatic DMPESI peaks are shifted and broadened relative to neat DMPESI. These species have chemical shifts which differ substantially from neat DMPESI and we therefore attribute them to an intermediate phase and/or products of a more complex chemical reaction involving DMPESI and $FAPbI_3$ films. We also note that there are multiple FA local environments, confirming the complex composition of the drop-cast material. While the phase composition of the drop-cast material does not correspond to that made using spin-coating, we use it as a qualitative indication that DMPESI interacts with $FAPbI_3$ and undergoes a chemical transformation in its presence. The ^1H results on spin-coated films evidence that the resulting species interact directly with the perovskite phase. We interpret these results as the underlying chemical reason for the improved stability of DMPESI-$FAPbI_3$. That said, we note that the chemical reactivity of DMPESI and the identity of the resulting species is more complex and merits further investigations.

Supplementary Table 4. NMR acquisition and processing parameters used for the different materials reported here.

Sample	Nucleus, pulse sequence	T_1 [s]	Recycle delay [s]	# of scans	Magnetic field [T]	MAS rate [kHz]	Apodization [Hz]
Neat DMPESI	^{1}H, Hahn echo	2.1 (^{1}H)	5	4	23.5	50	0
spin-coated passivated film	^{1}H, Hahn echo	26.5 (^{1}H)	150	4	23.5	50	0
Neat DMPESI	^{13}C, CP	1.7 (^{1}H)	2	1520	14.1	12	0
drop-cast passivated film	^{13}C, Hahn echo	n/d (^{13}C)	10	7296	14.1	12	100
drop-cast passivated film	^{13}C, CP	0.6, 11.3 (biexponential) (^{1}H)	10	460	14.1	12	100

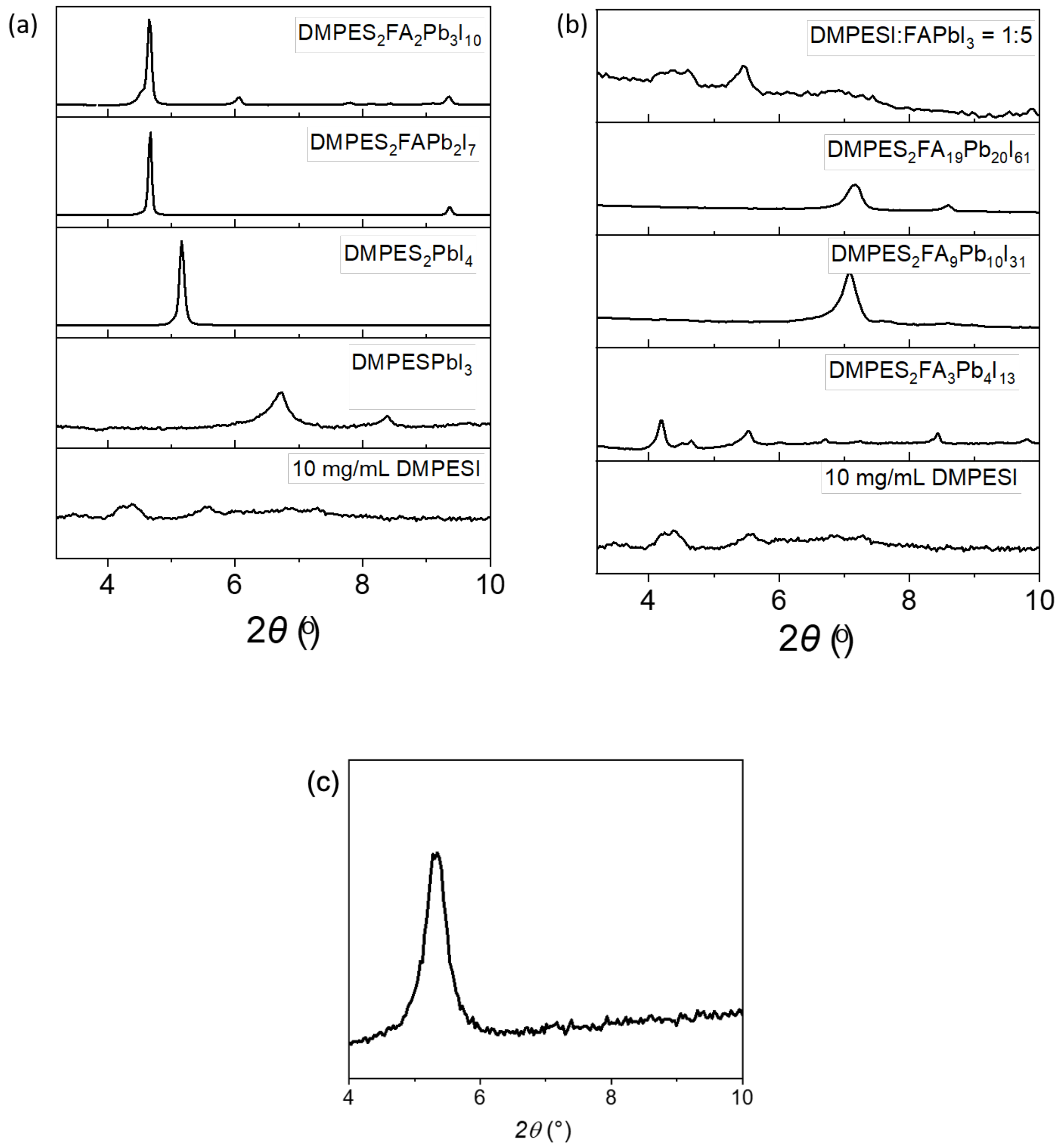

Supplementary Figure 14. **XRD patterns of different DMPESI-FA lead iodides and PEAI.** (a), (b) XRD patterns of different DMPESI-FA lead iodides prepared using stoichiometries corresponding to the nominal stoichiometries of Ruddlesden-Popper phases, (c) XRD pattern of PEAI (10mg/mL) treated perovskite film.

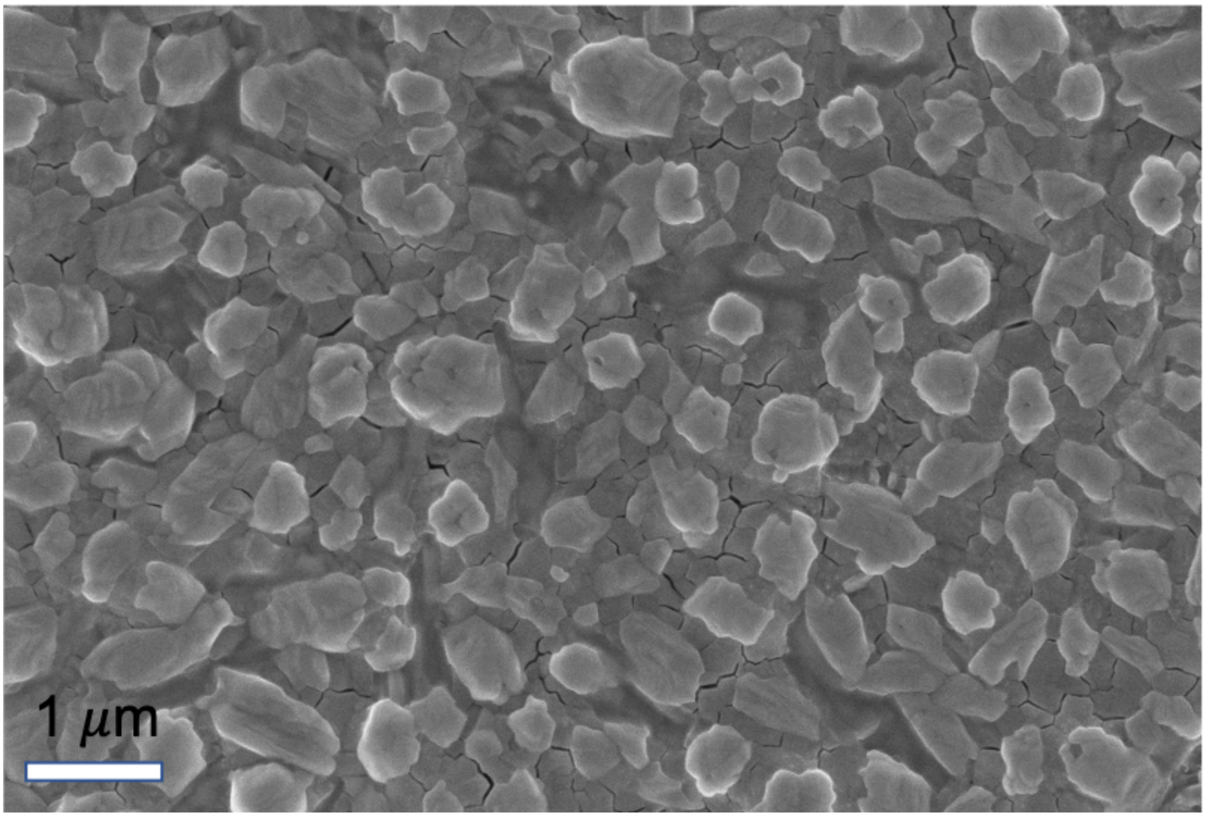

Supplementary Figure 15. **Top-view SEM images of the mixture film**. The ratio of DMPESI and FAPbI3 is 1:5.

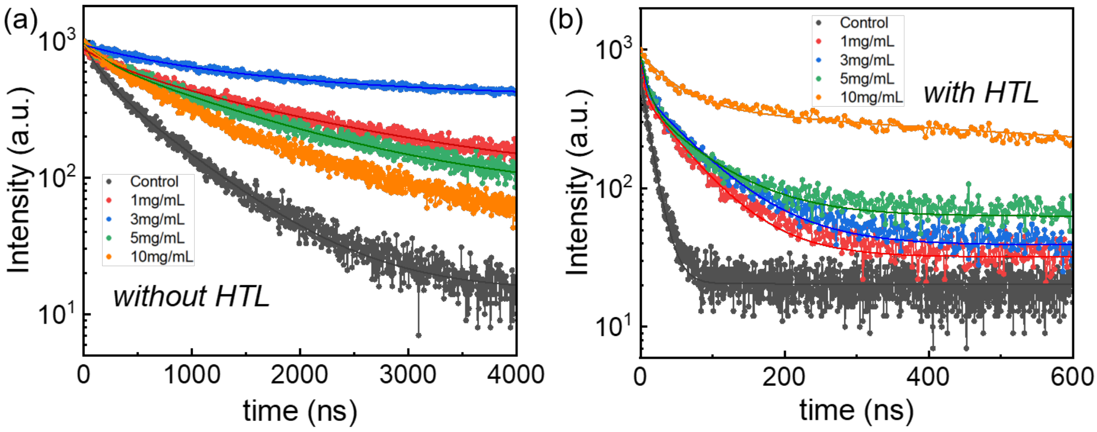

Supplementary Figure 16. TRPL measurements. TRPL for the layer structure of (a) glass/perovskite/with and without DMPESI treatment and (b) glass/perovskite/with and without DMPESI treatment/HTL. Detals of the fits are provided in Supplementary Note 4 below.

Supplementary Note 4

To gain a deeper look into the effect of DMPESI on the charge carrier dynamics in PSCs, we analyzed the TRPL-measurements in Supplementary Fig. 16 and in more detail by calculating the carrier density-dependent differential lifetime. This approach was recently proposed by Kirchartz et al[17]. as an effective method to disentangle between different mechanisms affecting PL-decay, such as different recombination mechanisms, charge carrier extraction as well as capacitive effects[17]. The differential lifetime τ_{TPL} is defined as:

$$\tau_{TPL} = \left(\frac{1}{m}\frac{d\ln(I_{PL})}{dt}\right)^{-1} \quad (2)$$

where m = 1 for low-level injection and m = 2 for high-level injection, I_{PL} is PL intensity (which can be normalized) and t – time after excitation.

By knowing the excitation fluence, the total absorbed number of absorbed photons at the $t = 0$ can be found using:

$$G = \alpha\phi_0 \exp{(-\alpha z)} \quad (3)$$

where ϕ_0 denotes photon flux at the sample surface (which is found from laser fluence), α – perovskite absorption coefficient and z – depth. Integrating G over the penetration depth yields total absorbed photon density, yielding the initial excess carrier density Δn_0. Under high-level injection regime the maximum quasi-Fermi level splitting (ΔE_F) can be calculated via:

$$\Delta E_{F,max} = \frac{kT}{q}\ln\left(\frac{\Delta {n_0}^2}{{n_i}^2}\right) \quad (4)$$

where n_i is intrinsic charge carrier density. Since photon flux depends exponentially on the $\Delta E_F(t)$, it can be plotted via proportionality:

$$\Delta E_F(t) \propto \ln(I_{PL}(t))\,\frac{kT}{q} \quad (5)$$

Hence allowing to plot differential lifetime as a function of QFLS or excess carrier density Δn. For more details we would like to refer to the original work by Kirchartz et al[17].

Supplementary Fig 17 shows the $\tau_{TPL}(t)$ and $\tau_{TPL}(\Delta E_F)$ of the control perovskite film and the one treated with DMPESI (3 mg/mL). Firstly, we note a difference in the τ_{TPL} at short time-scales, which is determined by the radiative recombination rate. More importantly, the τ_{TPL} on a µs-scale, which is mainly influenced by the defect-induced Shockley-Read-Hall (SRH) recombination, is enhanced by almost one order of magnitude in the DMPESI-sample in comparison to that of the control perovskite film. In the absence of higher order recombination mechanisms (radiative and Auger), the τ_{TPL} would be 16 µs in DMPESI-treated layers and 3.26 µs in control perovskite films, effectively demonstrating strong suppression of surface defect states in perovskite layer upon introduction of DMPESI.

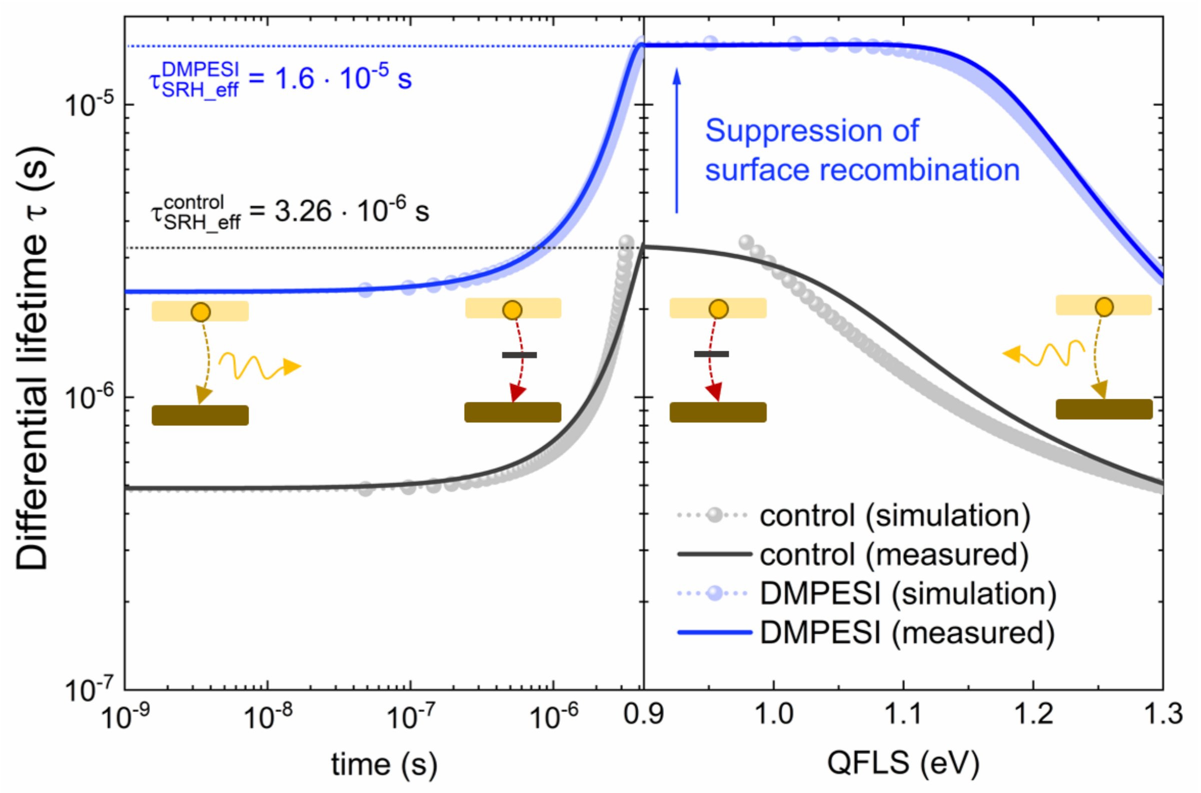

Supplementary Figure 17. Differential lifetime of control and DMPESI-treated films as function of time after excitation and QFLS. The rational fits of the experimentally measured PL-decays, exhibit a strong correlation with our simulated PL decays. The illustrations in the middle demonstrate that the radiative processes dominate at early times, while the defect-induced SRH recombination affects the decay primarily in µs-range.

To quantify the reduction of non-radiative surface recombination due to DMPESI-passivation, we implement a numerical simulation to fit the PL decays and extract the properties related to non-radiative recombination. To describe the change in excess charge-carrier concentration (Δn) over time due to recombination processes (except Auger recombination) present in a semiconductor, the following rate equation can be used:

$$\frac{d\Delta n}{dt} = B_{rad}(np - n_i^2) - \frac{np - n_i^2}{\tau_p n + \tau_n p} \qquad (6)$$

where B_{rad} is the radiative recombination coefficient, n and p are the total electron and hole concentrations, respectively, and τ_p and τ_n are hole and electron lifetimes, respectively. Note that here we assumed that the concentration gradients equilibrate fast enough so that drift and diffusion currents can be neglected. In the case of undoped semiconductors, like lead-halide perovskites we can assume approximately equal concentrations of hole and electrons ($n = p$), simplifying the Equation (6) to:

$$\frac{d\Delta n}{dt} = B_{rad} n(t)^2 - \frac{n(t)}{(\tau_p + \tau_n)} = B_{rad} n(t)^2 - \frac{n(t)}{\tau_{SRH}} \qquad (7)$$

In order to account for a surface non-radiative recombination, the total carrier lifetime τ_{SRH} can be considered as:

$$\tau_{SRH} = \left(\frac{1}{\tau_{bulk}} + \frac{1}{\tau_{surf}}\right)^{-1} \quad (8)$$

where the τ_{bulk} is the lifetime limited by the SRH recombination in the bulk and τ_{surf} is the lifetime limited by the surface recombination, which can be described as

$$\tau_{surf} = \frac{d}{S} \quad (9)$$

where and S is surface recombination velocity (assuming it is equal for electrons and holes). In this case we assume that one surface (perovskite/glass interface) is perfectly passivated.

Analytically solving Equation (7) yields:

$$\Delta n(t) = \frac{\Delta n_0 e^{-t/\tau_{SRH}}}{1+\Delta n_0 B_{rad}\tau_{SRH}(1-e^{-t/\tau_{SRH}})} \quad (10)$$

If only the SRH-recombination were present, one could fit the analytical solution of the Equation (9) to the experimentally obtained mono-exponential decay to extract the τ_{SRH}. However, the mono-exponential model based on Equation (10) does not match the experimentally observed PL-decays of our samples which might be due to other recombination mechanisms present in perovskite-based materials outside of the classic SRH-theory. To fit our model more accurately and qualitatively compare the SRH lifetimes of passivated and non-passivated films we modify the Equation (10) to a bi-exponential model described by:

$$\Delta n(t) = \frac{A_1 \Delta n_0 e^{-t/\tau_1} + A_2 \Delta n_0 e^{-t/\tau_2}}{1+\Delta n_0 B_{rad}[A_1\tau_1(1-e^{-t/\tau_1})+A_2\tau_2(1-e^{-t/\tau_2})]} \quad (11)$$

where A_1 and A_2 are the weight coefficients of two-time constants: τ_1 (initial fast decay) and the second time constant τ_2 describing longer decay. Since the recombination at longer times is mainly influenced by the first-order SRH-recombination, we denote τ_2 as τ_{SRH}. –.

Using the values of τ_{SRH} obtained from the differential lifetime we observe a strong correlation between our model and the experimental PL-decays (Supplementary Fig. 17) when the S_{DMPESI} = 4 cm/s, while the $S_{control}$ = 65 cm/s, highlighting that the surface recombination velocity is reduced by almost 16 times, when perovskite films are treated with DMPESI. Assuming that the nature of energy traps is the same in both: treated and untreated samples, the trap density in control samples are also expected to be 16 times higher than in DMPESI-ones. Hence, DMPESI can be used as an effective passivation layer to suppress non-radiative surface recombination.

Next, we use the differential lifetime approach to gain a deeper understanding of the perovskite/HTL interface in the presence of DMPESI. Since the charge extraction from perovskite to HTL occurs on 10^{-12} – 10^{-9} time-scales, the extraction efficiency can be qualitatively compared by looking at the corresponding differential lifetimes in Fig. 3b, 3c. The

fastest charge extraction occurs in sample without DMPESI and the slowest for the sample with the thickest DMPESI layer. However, at t ≈ 10^{-7} s, the PL of the perovskite/HTL sample decreases to a noise value, effectively meaning complete charge carrier depopulation. In contrast, all the DMPESI samples exhibit a much longer decay with τ_{TPL} in a µs-range.

Importantly, the τ_{SRH} is prolonged from 2 to 3.5 µs when the DMPESI concentration is slightly increased from 1 mg/mL to 3 mg/mL. However, increasing the DMPESI concentration (and therefore layer thickness) further does not result in better suppression of non-radiative recombination, as evident by the same τ_{TPL} at between 10^{-7} – 10^{-8} s. Instead, we notice a pronounced prolongation of τ_{TPL} at high QFLS, which is a sign of poorer charge carrier depopulation at shorter times, normally caused by a lower charge extraction efficiency.

These results suggest that moderate quantities of DMPESI (<5 mg/mL) strongly reduce the non-radiative surface recombination at perovskite/HTL interface, while simultaneously allowing for an efficient charge extraction. Although thicker DMPESI layers also effectively suppress non-radiative recombination, this benefit might become outweighed by significantly worse charge extraction, possibly, due to lower charge carrier conductivity and poor band alignment. To visualize the effect of DMPESI layer thickness on charge carrier dynamics at perovskite/HTL interface we employed a 1D drift-diffusion SCAPS simulation of a perovskite/passivation/spiro-OMeTAD stack with generic values for the passivation layer, assuming the properties of DMPESI are similar to other passivation layers reported in perovskite literature (e.g. higher energy bandgap, lower charge carrier mobility). All parameters of the simulation can be found in Supplementary Table 5.

SCAPS is an open-source 1D drift-diffusion simulation tool, that can be obtained from https://users.elis.ugent.be/ELISgroups/solar/projects/scaps upon the request from the developers Marc Burgelman et al.

Supplementary Table 5: Parameters of the perovskite/spiro-OMETAD and Perovskite/passivation/spiro-OMeTAD simulation via SCAPS

Property	PVSK/spiro-OMeTAD)	PVSK/passivation/ spiro-OMeTAD
Perovskite layer thickness	650 nm	
E_g perovskite	1.47 eV	
Electron affinity	4 eV	
Dielectric permittivity (relative)	6.6	
Electron/hole mobility	10 cm^2/Vs	
Trap density N_t in the bulk	10^{13} cm^{-3}	

E_t (above E_V)	600 meV	
Capture cross section at the GBs	10^{-15} cm^2	
Effective electron density of states ($N_{c/v}$) – perovskite	$1.2*10^{19}$ cm^{-3}	
passivation layer thickness	-	**1 nm, 3 nm, 5 nm and 10 nm**
Surface recombination of electrons/holes ($S_{e,h}$) at Perovskite/passivation interface	-	**40 cm/s (1 nm), 4 cm/s (3 nm), 4 cm/s (5 nm), 4 cm/s (10 nm)**
E_g passivation	-	1.66 eV
Electron affinity – passivation	-	3.63 eV
Dielectric permittivity (relative) of passivation layer	-	30
Electron/hole mobility – passivation	-	10^{-4} cm^2/Vs
Effective electron density of states ($N_{c/v}$) – passivation	-	10^{18} cm^{-3}
Surface recombination of electrons/holes ($S_{e,h}$) at Perovskite/spiro-OMeTAD interface	120 cm/s	0 cm/s
Spiro-OMeTAD thickness	125 nm	
E_g Spiro-OMeTAD	3.06 eV	
Electron affinity – Spiro-OMeTAD	2.05 eV	
Dielectric permittivity (relative) of Spiro-OMeTAD layer	3	
Electron/hole mobility – Spiro-OMeTAD	10^{-4} cm^2/Vs	
Effective electron density of states ($N_{c/v}$) – Spiro-OMeTAD	$2.2*10^{18}$ cm^{-3}	
Acceptor density of states (N_A) – Spiro-OMeTAD	$1.3*10^{18}$ cm^{-3}	

Simulation results in Supplementary Fig. 18 demonstrate a rise in the net charge density at deeper z, which peaks at the perovskite/DMPESI interface due to strong charge accumulation at the surface. The charge density peak increases with higher DMPESI thickness (d^{DMPESI}) due to higher accumulation of charge carriers (in this case holes). This effect creates a narrow space-charge region, slightly reducing the hole transport across perovskite (HTL interface). On the other hand, the surface recombination velocities at perovskite/HTL and perovskite/DMPESI interfaces also affect the final extracted hole current J_h. Due to the high $S_{control}$, the $J_h^{control}(z > 650\ nm)$ is lower than the $J_h^{3\ mg/mL}$ and $J_h^{5\ mg/mL}$ and nearly equal to the $J_h^{1\ mg/mL}$ (due to slightly lower $\tau_{SRH}^{1\ mg/mL}$, as seen from the Fig. 3b, 3c). We note that this

trend in $J_h(d^{\mathrm{DMPESI}})$ agrees well with the way how the short-circuit current density (J_{SC}) of the manufactured PSCs changes with larger d^{DMPESI} (Fig. 3b, 3c), underlining the validity of our simulation. Based on this simulation, we propose a similar explanation of the influence of DMPESI on the hole extraction, where the 3mg/mL DMPESI layer strongly suppresses non-radiative recombination at the perovskite/HTL surface, while simultaneously being thin enough not to build-up large electric field and not to inhibit the hole-extraction.

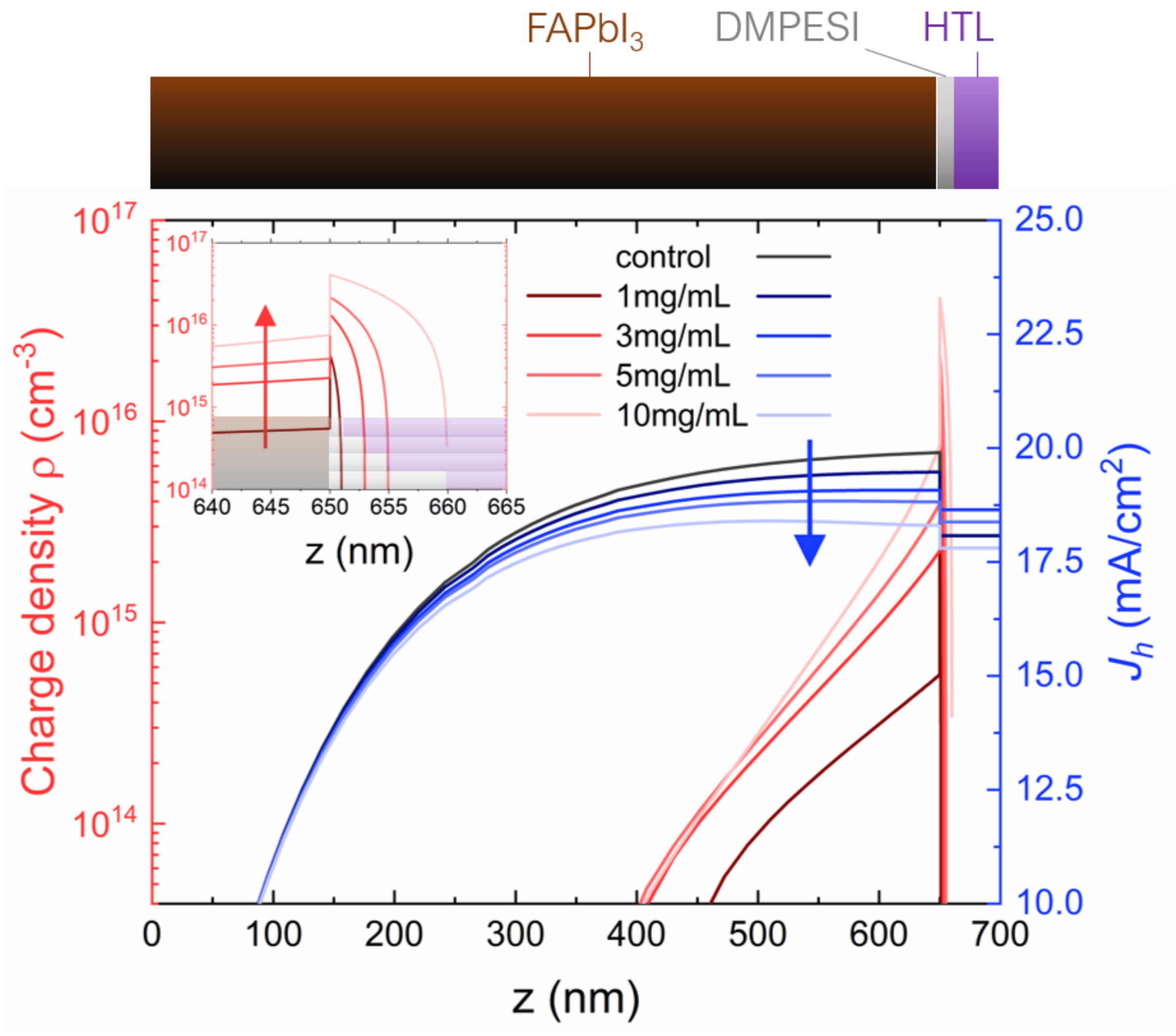

Supplementary Figure 18. SCAPS simulation. SCAPS simulation of charge density ρ and hole current density in samples with different passivation layer thicknesses (DMPESI solution concentrations) as function of sample depth under illumination. The inset shows the peak of the charge density at the perovskite/DMPESI interface, highlighting excessive charge accumulation at this interface which inhibits effective hole extraction from perovskite.

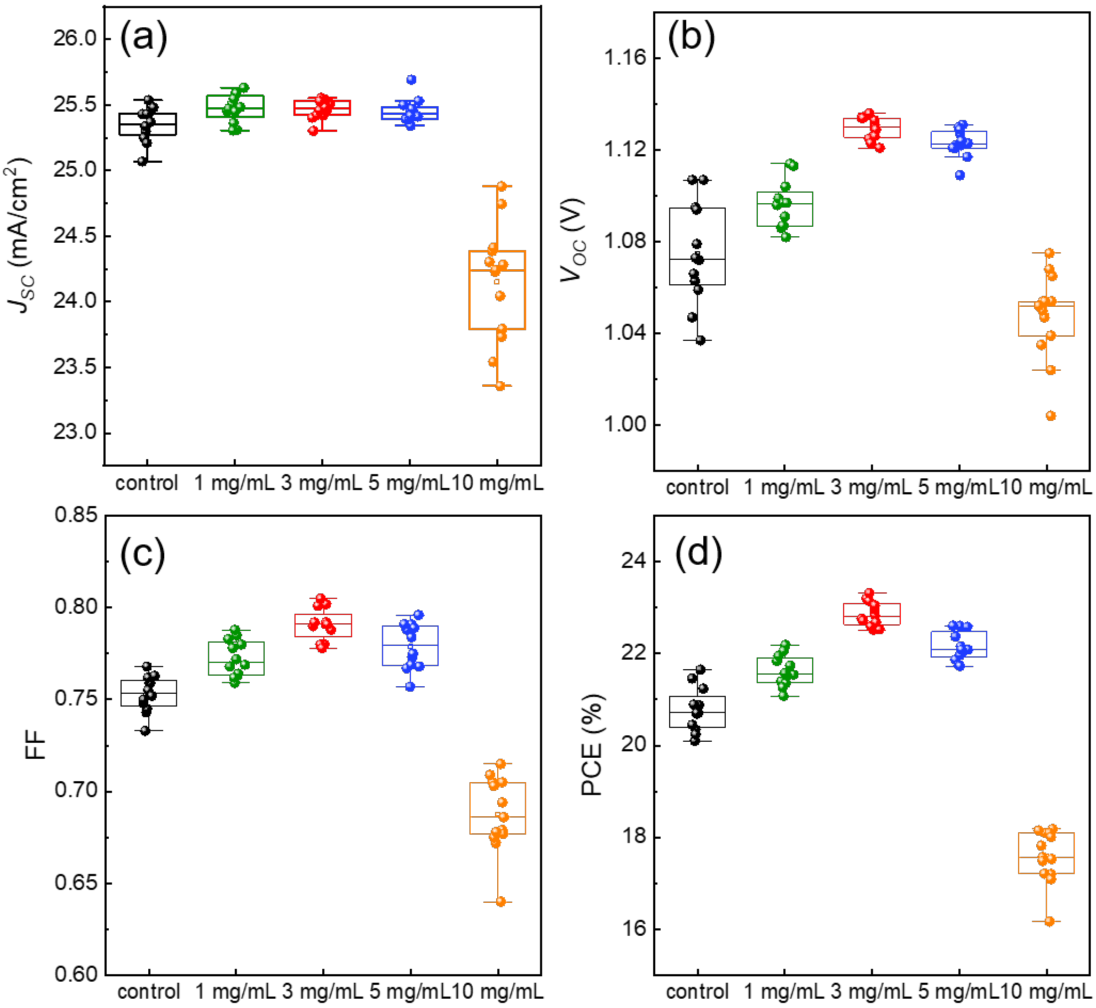

Supplementary Figure 19. Statistical box charts for the photovoltaic parameters. (a) J_{SC}, (b) V_{OC}, (c) FF, and (d) PCE of the 15 devices for each condition without and with DMPESI treatment of different concentrations (1mg/mL, 3mg/mL, 5mg/mL, 10 mg/mL). The box plots display the mean, median line, 25-75% box limits and the whiskers represent the minimum and maximum values. Each dot represents one device.

Supplementary Table 6 Photovoltaic parameters of champion control and post-treated with various concentrations of DMPESI devices.

	Jsc **(mA/cm^2)**	**Voc (V)**	**FF**	**PCE (%)**
**Control**	25.42	1.107	0.769	21.65
**1mg/mL**	25.59	1.114	0.779	22.19
**3mg/mL**	25.53	1.136	0.805	23.32
**5mg/mL**	25.50	1.121	0.791	22.61
**10mg/mL**	24.33	1.052	0.709	18.15

Supplementary Table 7. Photovoltaic parameters of DMPESI/PEAI treated PSCs under various conditions.

	**Anealing temperature**	***J*****sc (mA/cm^2)**	***V*****oc (V)**	**FF**	**PCE (%)**
**DMPESI**	100 °C	25.60±0.12	1.132±0.007	0.792±0.013	22.97±0.33
**DMPESI**	60 °C	25.53±0.24	1.121±0.009	0.774±0.018	22.15±0.64
**DMPESI**	25 °C	25.47±0.32	1.101±0.012	0.772±0.019	21.64±0.81
**DMPESI**[a]	100 °C	25.53±0.17	1.127±0.004	0.792±0.015	22.79±0.52
**PEAI**	100 °C	25.43±0.21	1.124±0.012	0.778±0.025	22.22±0.77

a. The annealing time is 5 min, ten devices for each condition.

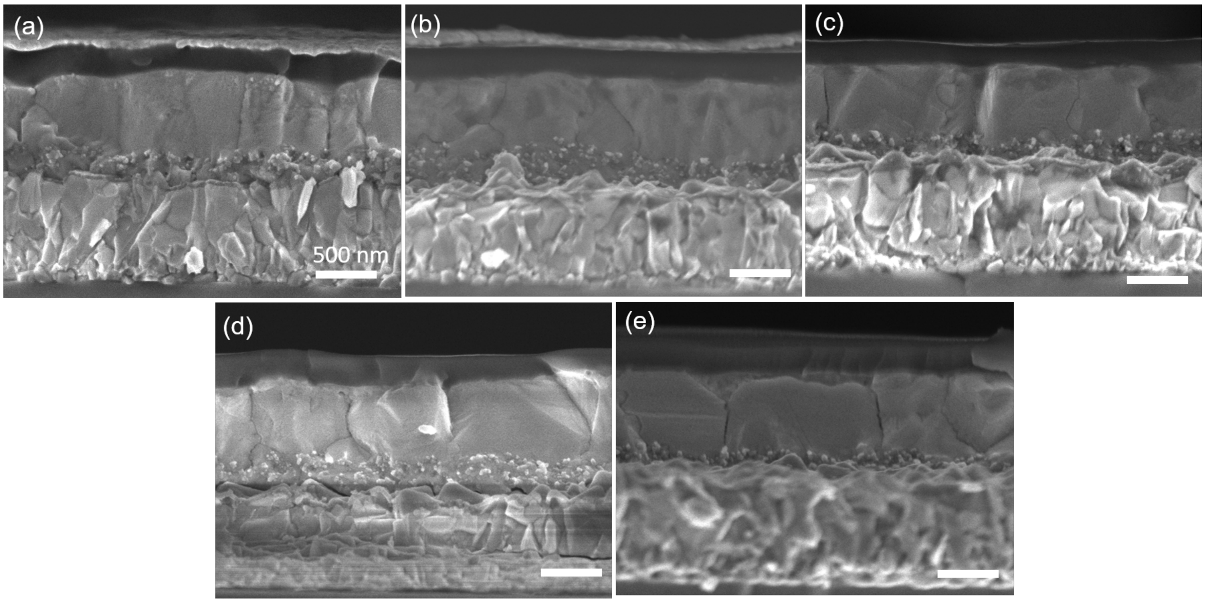

Supplementary Figure 20. **Cross-sectional SEM images.** (a) control device and devices treated with (b) 1 mg/ml, (c) 3 mg/ml, (d) 5 mg/ml, (e) 10 mg/ml DMPESI.

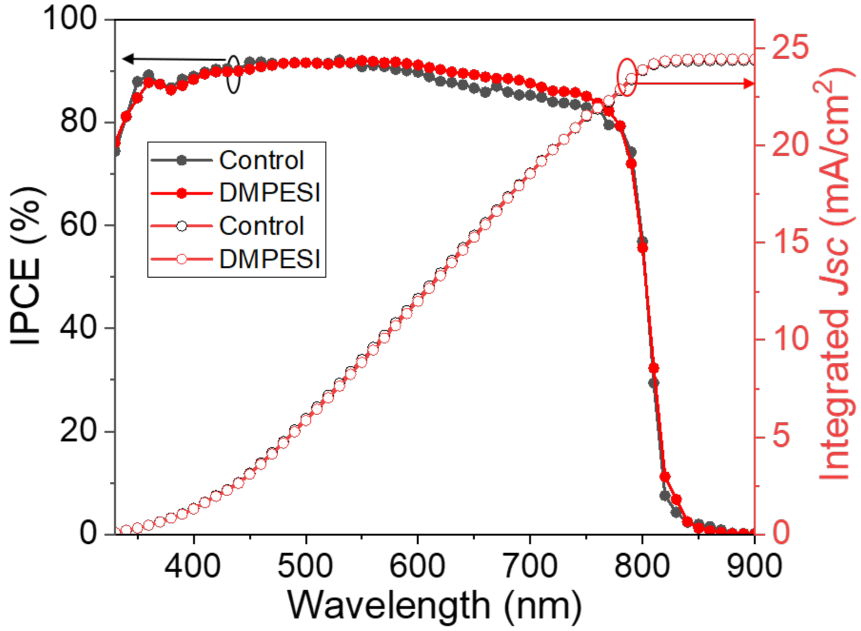

Supplementary Figure 21. **IPCE and integrated *Jsc***. IPCE and integrated photocurrent density of devices with and without DMPESI (3 mg/mL) treatment.

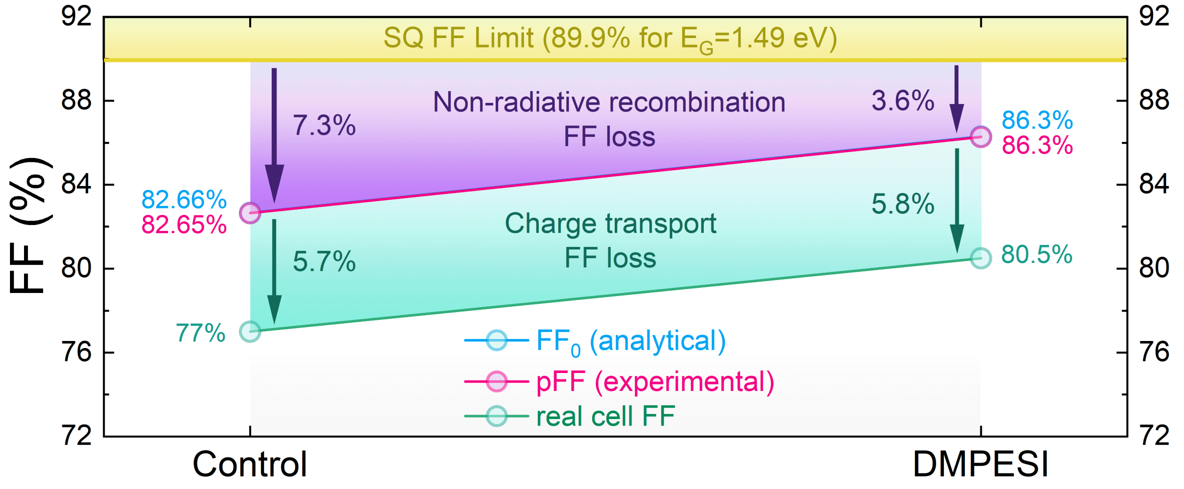

Supplementary Figure 22. FF loss analysis. FF loss analysis showing the differences between the maximum FF limited by the radiative recombination (SQ FF limit), the measured pseudo-FF (and calculated FF0), describing the cell FF in the absence of charge-transport losses and the measured cell FF. As can be clearly seen, the improvement in FF by introducing the DMPESI layer (~3.5%) comes from reduction of the non-radiative recombination FF loss. The analytically calculated FF in the absence of the charge transport loss – FF_0 was calculated according to the expression proposed by Green et al[18].

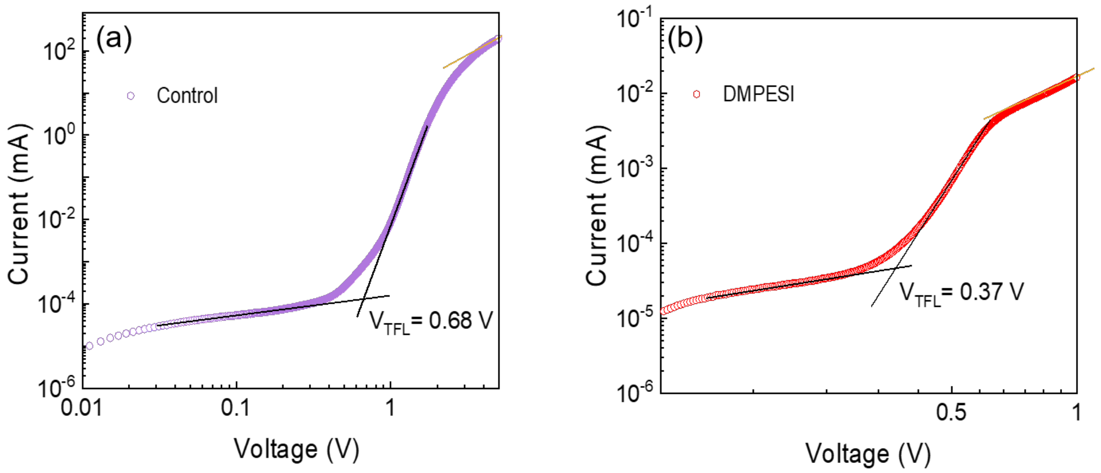

Supplementary Figure 23. **Space-charge-limited-current (SCLC) measurements.** I-V curves of the SCLC measurements based on the electron-only devices with (a) control perovskite and (b) DMPESI. SCLC measurements carried out by measuring the IV characteristics. The switch between the ohmic regime (determined by the background charge carrier concentration) and the Mott-Gurney regime (determined by the buildup of the space-charge and energy traps) happens at the trap-filled voltage level (V_{TFL}) at which the trap-filling process is assumed to be completed. Thus, a lower V_{TFL} in DMPESI-treated samples suggests a lower number of surface traps of perovskite absorbers.

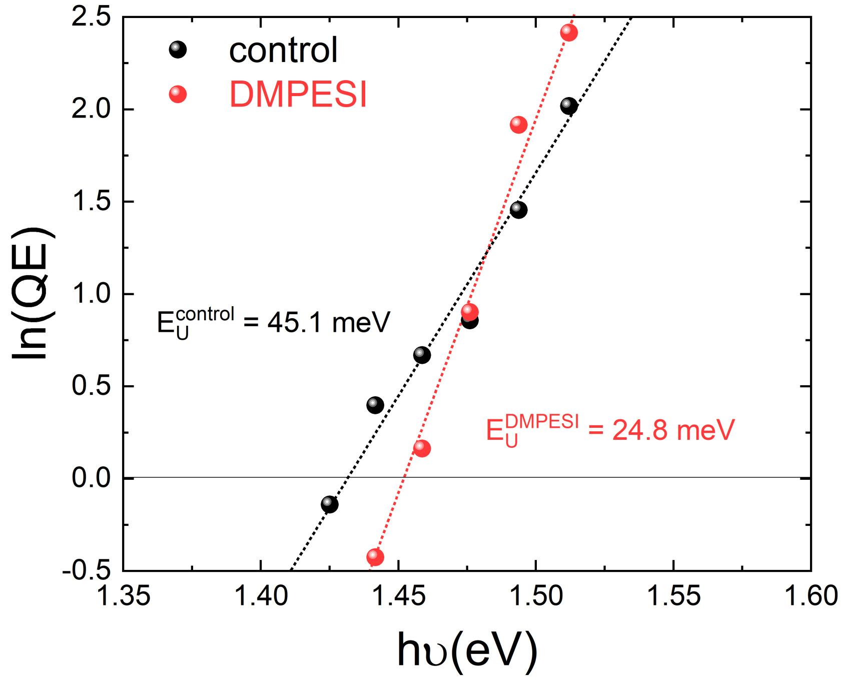

Supplementary Figure 24. Devices Urbach energy. Urbach energy of the control and DMPESI-treated samples calculated from the quantum efficiency (QE) measurements. Urbach energy of the control and DMPESI-treated samples extracted from the quantum efficiency (QE) measurements, by linearly fitting the slope of the ln(QE) plot at the intercept.

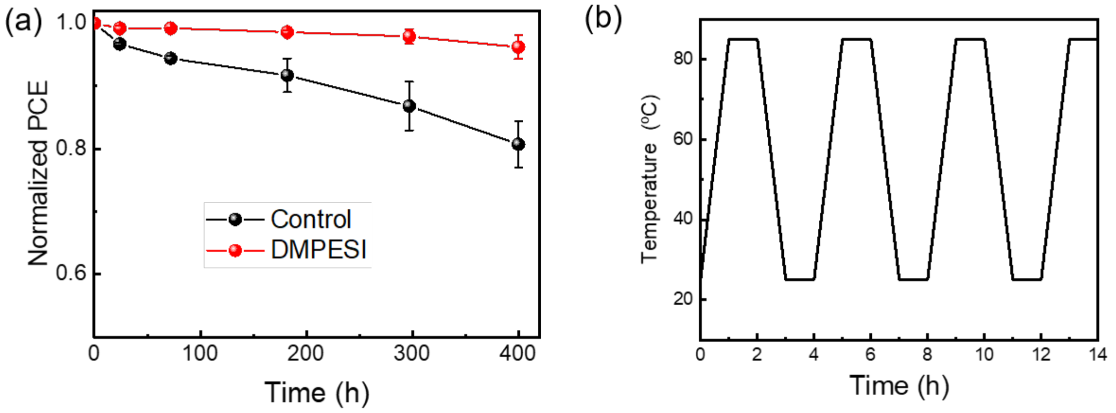

Supplementary Figure 25. **PSCs under thermal stress and the procedure of temperature cycling.** (a)Normalized PCE tracking of the unencapsulated control and target PSCs after 400 h under thermal stress at 60 °C under R.H. of less than 30%, 5 devices for each condition, the initial device PCEs are 22.97 ± 0.29% (DMPESI) and 20.96 ± 0.32 % (control). Data are presented as mean values ± SEM. (b) the temperature cycling (25 °C – 85 °C) procedure of encapsulated devices.

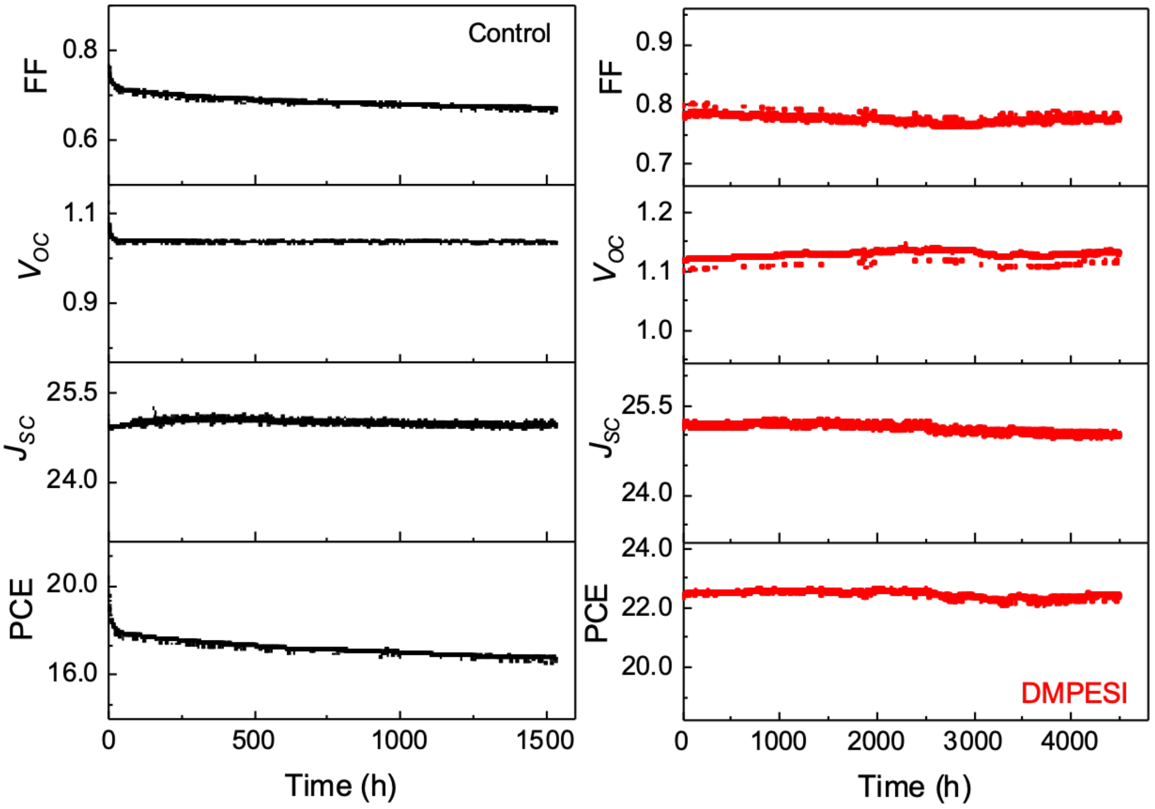

Supplementary Figure 26. **Photovoltaic parameters of devices under MPPT.** Photovoltaic parameters of FF, V_{OC}, J_{SC} and PCE the control and target devices under MPPT with continuous one sun illumination under N_2 flow at room temperature.

Supplementary Table 8. Summary of operational stabilities of highly efficient (PCE>22%) devices under at continuous one-sun illumination

No.	3D perovskite	Architecture	Champion PCE	Ageing time	Retaining PCE	Ref.
1	$FAPbI_3$	n-i-p	23.7%	600 h	>90%	*19*
2	$FAPbI_3$	n-i-p	22.98%	971 h	92.1%	*20*
3	$FAPbI_3$	n-i-p	24.1%	500 h	90%	*21*
4	$FAPbI_3$	n-i-p	25.17%	400 h	>90%	*22*
5	$FAPbI_3$	n-i-p	23.9%	1000 h	~95%	*23*
6	$FAPbI_3$	n-i-p	25.6%	450 h	85%	*24*
7	$FAPbI_3$	n-i-p	23.1%	500 h	94%	*25*
8	$FAPbI_3$	n-i-p	25.2%	500 h	80%	*26*
9	$FAPbI_3$	n-i-p	24.02%	500h	89%	*27*
10	$FAPbI_3$	n-i-p	25.8%	500h	90%	*28*
11	$FAPbI_3$	n-i-p	25.1%	700h	90%	*29*
12	$(FAPbI_3)_{0.92}(MAPbBr_3)_{0.08}$	n-i-p	23.4%	500h	85%	*30*
13	$MA_{0.1}FA_{0.85}Cs_{0.05}PbI_{2.9}Br_{0.1}$•$0.05PbI_2$	n-i-p	23.5%	500h	95%	*31*
14	$(FAPbI_3)_{0.95}(MAPbBr_3)_{0.05}$	n-i-p	24.35%	1620h	98%	*32*
15	$(FAPbI_3)_{0.95}(MAPbBr_3)_{0.05}$	n-i-p	24.3%	500h	>95%	*33*
16	$Cs_{0.1}(FA_{0.92}MA_{0.08})_{0.9}Pb(I_{0.9}Br_{0.04}Cl_{0.06})_3$	n-i-p	22.29%	1235h	95%	*34*
17	$(FAPbI_3)_{0.94}(MAPbBr_3)_{0.06}$	n-i-p	23.0%	2000h	85%	*35*
18	$MA_XFA_{1-X}PbI_3$	n-i-p	23.9%	2000h	97%	*36*
19	$MA_XFA_{1-X}PbI_3$	n-i-p	24.04%	2000h	95%	*37*
20	$(FAPbI_3)_{0.95}(MAPbBr_3)_{0.05}$	n-i-p	23.3%	1370h	95%	*38*

21	$MA_{0.05}FA_{0.90}Cs_{0.05}PbI_{2.95}Br_{0.05}\cdot 0.10PbI_2$	n-i-p	23.3%	500h	>90%	*39*
22	$MA_{0.1}FA_{0.85}Cs_{0.05}PbI_{2.9}Br_{0.1}\cdot 0.05PbI_2$	n-i-p	23.1%	450h	>95%	*9*
23	$MA_{0.1}FA_{0.85}Cs_{0.05}PbI_{2.9}Br_{0.1}\cdot 0.05PbI_2$	n-i-p	23.5%	600h	>92%	*7*
24	$MA_{0.1}FA_{0.85}Cs_{0.05}PbI_{2.9}Br_{0.1}\cdot 0.05PbI_2$	n-i-p	23.9%	500h	>96%	*40*
25	$(FAPbI_3)_{0.95}(MAPbBr_3)_{0.05}$	n-i-p	23.7%	300h	80%	*41*
26	$MA_XFA_{1-X}PbI_{3-y}Br_y$	n-i-p	23.48%	500h	>90%	*42*
27	$MA_XFA_{1-X}PbI_{3-y}Br_y$	n-i-p	22.9%	200h	86%	*43*
28	$MA_XFA_{1-X}PbI_{3-y}Br_y$	n-i-p	23.82%	200h	83.5%	*44*
29	$MA_XFA_{1-X}PbI_{3-y}Br_y$	n-i-p	23.24%	180h	91%	*45*
30	$MA_{0.03}FA_{0.97}PbI_{2.81}Br_{0.09}$	n-i-p	23.4%	1440h	87%	*46*
31	$MA_{0.1}FA_{0.85}Cs_{0.05}PbI_{2.9}Br_{0.1}\cdot 0.05PbI_2$	n-i-p	23.57%	1500h	96%	*47*
32	$MA_{0.1}FA_{0.85}Cs_{0.05}PbI_{2.9}Br_{0.1}$	n-i-p	24.7%	1000h	90%	*48*
33	$Cs_{0.05}FA_{0.88}MA_{0.07}PbI_{2.56}Br_{0.44}$	n-i-p	23.36%	250h	98.7%	*49*
33	$FA_{0.851}MA_{0.149}PbI_{2.552}Br_{0.448}$	n-i-p	23.16%	1000h	90%	*50*
34	$MA_XFA_{1-X}PbI_{3-y}Br_y$	n-i-p	24%	1000h	92%	*51*
35	$Cs_{0.05}(MA_{0.10}FA_{0.85})Pb(I_{0.90}Br_{0.10})_3$	n-i-p	24.5%	2000h	>99%	*52*
36	$Cs_xMA_yFA_{1-x-y}PbI_{3-z}Br_z$	n-i-p	23.92%	1100	>90%	*53*
37	$MA_{0.6}FA_{0.4}PbI_3$	p-i-n	23.6%	550h	100%	*54*
38	$MA_{0.14}FA_{0.81}Cs_{0.05}PbI_{2.58}Br_{0.42}\cdot 0.03PbI_2$	p-i-n	22.74%	1000h	93%	*55*

40	$(FAPbI_3)_{0.95}(MAPbBr_3)_{0.05}$	p-i-n	24.3%	1000h	90.5%	*56*
41	$Cs_{0.05}(FA_{0.98}MA_{0.02})_{0.95}Pb(I_{0.98}Br_{0.02})_3$	p-i-n	25%	1500h	>98%	*57*
42	$Rb_{0.05}Cs_{0.05}MA_{0.05}FA_{0.85}Pb(I_{0.95}Br_{0.05})_3$	p-i-n	25.35%	2400h	87%	*58*
43	**$FAPbI_3$**	**n-i-p**	**23.3%**	**4509 h**	**~100%**	**Our work**

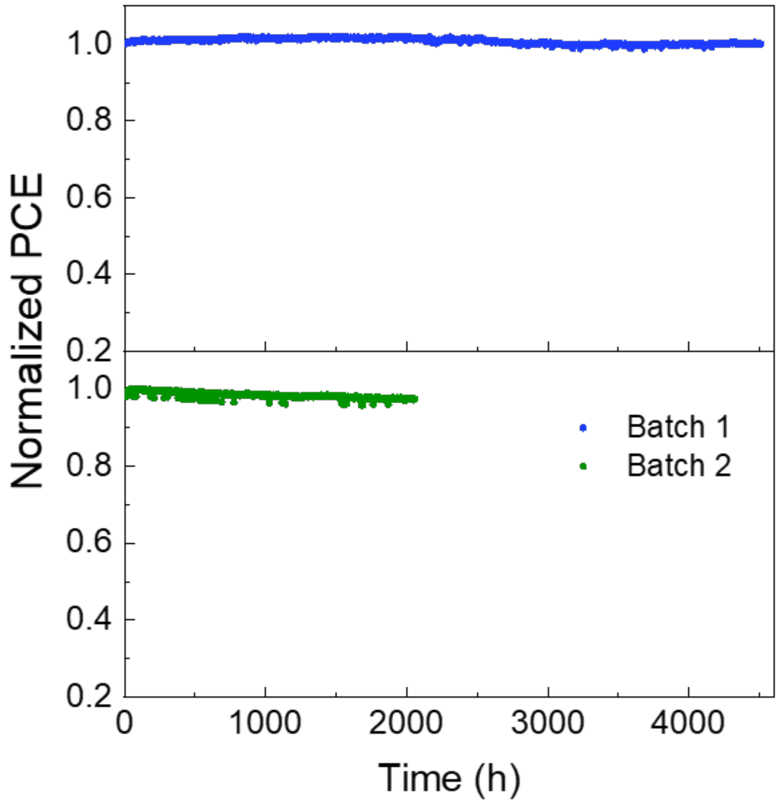

Supplementary Figure 27. Long-term operational stability of target devices from different batches. The devices were aged under MPPT with continuous one sun illumination under N_2 flow at room temperature. The initial PCEs of the devices are 22.57% (batch 1) and 22.36% (batch 2).

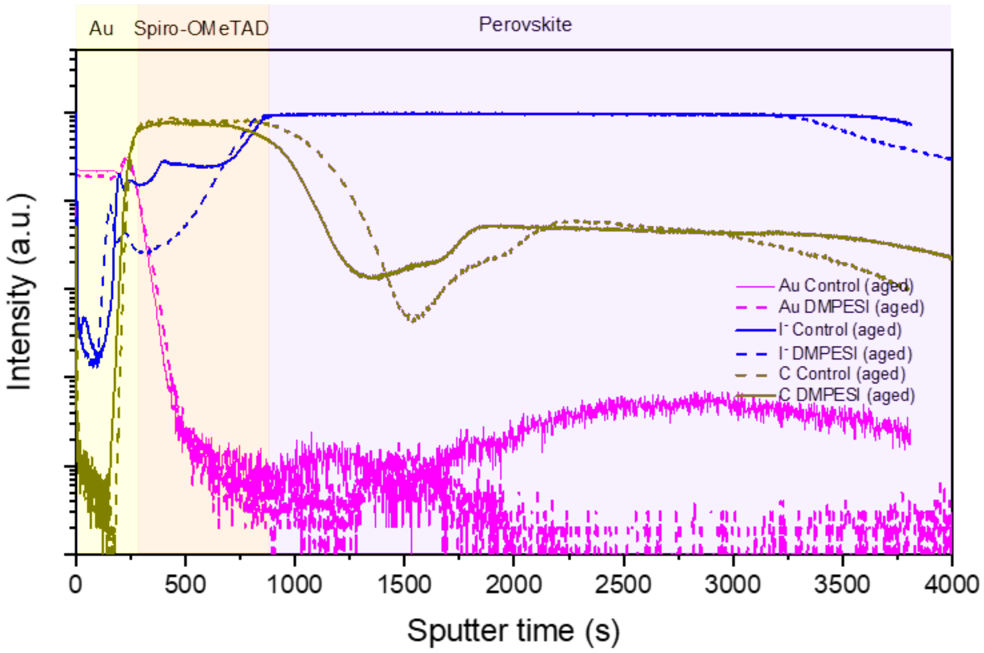

Supplementary Figure 28. ToF-SIMS depth profile analysis. ToF-SIMS depth profile analysis of control device (solid lines) and target device (dash lines) after 4509 h operational ageing.

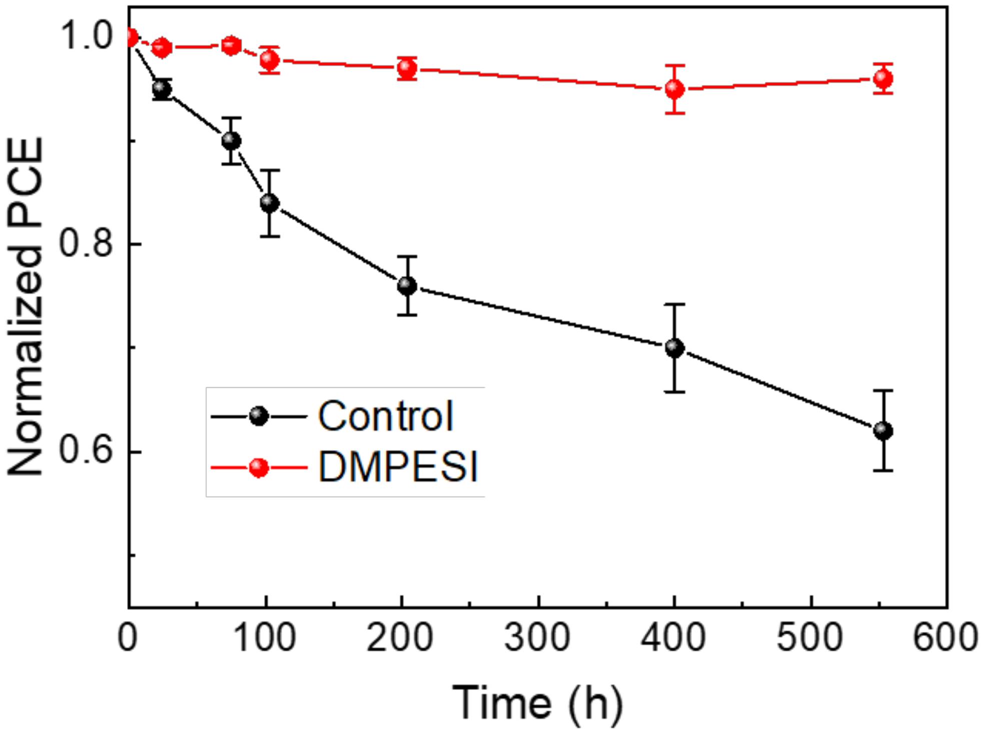

Supplementary Figure 29. Shelf-stability of the devices. Dark shelf-stability of unencapsulated control and DMPESI treated ($Cs_{0.05}MA_{0.1}FA_{0.85}PbI_{2.90}Br_{0.1}\cdot 0.05(PbI_2)$) PSCs in ambient condition at r.t. with R.H. around 20-40%, five devices for each condition, the initial device PCEs are 22.7 ± 0.32% (DMPESI) and 20.53 ± 0.52 % (control). Data are presented as mean values ± SEM.

Supplementary References

1. Frisch, M. J. et al. Gaussian 09, Revision A.02,. Gaussian, Inc., Wallingford CT, (2016).
2. Becke, A. D. J. Density-functional thermochemistry. III. The role of exact exchange. *Chem. Phys.* **98**, 5648−5652 (1993).
3. Hay. P. J. et al. *Ab initio* effective core potentials for molecular calculations. Potentials for the transition metal atoms Sc to Hg. *J. Chem. Phys*. **82**, 270 (1985).
4. Perdew, J. P. et al. Generalized gradient approximation made simple *Phys. Rev. Lett.* **77**, 3865-3868 (1996).
5. Stoumpos, C. C.; Malliakas, C. D.; Kanatzidis, M. G. Semiconducting Tin and Lead Iodide Perovskites with Organic Cations: Phase Transitions, High Mobilities, and Near-Infrared Photoluminescent Properties Inorg. Chem. **52**, 9019-9038 (2013).
6. Amat, A. *et al.* Cation-induced band-gap tuning in organohalide perovskites: interplay of spin–orbit coupling and octahedra tilting. *Nano Lett.* **14**, 3608-3616 (2014).
7. Suo, J. *et al*. Surface Reconstruction Engineering with Synergistic Effect of Mixed-Salt Passivation Treatment toward Efficient and Stable Perovskite Solar Cells, *Adv. Funct. Mater.,* **31**, 2102902, (2021).
8. Hassan, Y. *et al.* Ligand-engineered bandgap stability in mixed-halide perovskite LEDs. *Nature* **591**, 72–77 (2021).
9. Yang, B. *et al.* Outstanding passivation effect by a mixed-salt interlayer with internal interactions in perovskite solar cells. *ACS Energy Lett.* **5**, 3159-3167 (2020**)**.
10. Mahata, A. *et al.* Modulating Band Alignment in Mixed Dimensionality 3D/2D Perovskites by Surface Termination Ligand Engineering. *Chem. Mater*., **32**, 105−113 (2020).
11. Meggiolaro, E. *et al*. Energy Level Tuning at the $MAPbI_3$ Perovskite/Contact Interface Using Chemical Treatment. *ACS Energy Lett*., **4**, 2181−2184, (2019).
12. Yang, S. *et al.* Stabilizing halide perovskite surfaces for solar cell operation with wide-bandgap lead oxysalts. *Science*, **365**, 473–478, (2019).
13. Mosconi, E. *et al*. Intermolecular Interactions of A-Site Cations Modulate Stability of 2D Metal Halide Perovskites, ACS Energy Lett. **8**, 748–752 (2023).
14. Grimme S. *et al*. A consistent and accurate *ab initio* parametrization of density functional dispersion correction (DFT-D) for the 94 elements H-Pu. *J. Chem. Phys*. **132**, 154104 (2010).
15. Giannozzi, P. et al. Quantum espresso: a modular and open-source software project for quantum simulations of materials. *J. Phys.: Condens. Matter* **21**, 395502 **(**2009).
16. Kubicki, D. J. et al. NMR spectroscopy probes microstructure, dynamics and doping of metal halide perovskites. *Nat Rev Chem* **5**, 624–645 (2021).
17. Krückemeier, L. et al. Understanding transient photoluminescence in halide perovskite layer stacks and solar cells. *Adv. Energy Mater*. **11**, 2003489 (2021).
18. Green, M. A. Accuracy of Analytical Expressions for Solar Cell Fill Factors. *Solar Cells,* **7***,* 337-340 (1982).
19. Min, H. et al. Efficient, stable solar cells by using inherent bandgap of α-phase formamidinium lead iodide. *Science* **366**, 749-753 (2019).
20. Tan, S. et al. Shallow Iodine Defects Accelerate the Degradation of a-Phase Formamidinium Perovskite. *Joule* **4**, 2426-2442 (2020).
21. Hui, W. et al. Stabilizing black-phase formamidinium perovskite formation at room temperature and high humidity. *Science* **371**, 1359-1364 (2021).
22. Kim, G. et al. Impact of strain relaxation on performance of α-formamidinium lead iodide perovskite solar cells. *Science* **370**, 108-112 (2020).

23. Park, B.-W. et al. Stabilization of formamidinium lead triiodide α-phase with isopropylammonium chloride for perovskite solar cells. *Nat Energy*. **6**, 419-428 (2021).
24. Jeong, J. et al. Pseudo-halide anion engineering for α-$FAPbI_3$ perovskite solar cells. *Nature*, **592**, 381-385 (2021).
25. Lu, H. et al. Vapor-assisted deposition of highly efficient, stable black-phase $FAPbI_3$ perovskite solar cells. *Science*, **370**, eabb8985 (2020).
26. Yoo, J. J. et al. Efficient perovskite solar cells via improved carrier management. *Nature*, **590**, 587-593 (2021).
27. Guo, P. et al. Interfacial Embedding of Laser-Manufactured Fluorinated Gold Clusters Enabling Stable Perovskite Solar Cells with Efficiency Over 24%. *Adv. Mater.* **33**, 2101590 (2021).
28. Min, H. et al. Perovskite solar cells with atomically coherent interlayers on SnO_2 electrodes. *Nature*. **598**, 444-450 (2021).
29. Yun, H.-S. et al. Ethanol-based green-solution processing of α-formamidinium lead triiodide perovskite layers. *Nat Energy*. **7**, 828-834 (2022).
30. Yoo, J. J. et al. An interface stabilized perovskite solar cell with high stabilized efficiency and low voltage loss. *Energy Environ. Sci.*, **12**, 2192-2199 (2019).
31. Zhu, H. et al. Tailored Amphiphilic Molecular Mitigators for Stable Perovskite Solar Cells with 23.5% Efficiency. *Adv. Mater.* **32**, 1907757 (2020).
32. Jang, Y.-W. et al. Intact 2D/3D halide junction perovskite solar cells via solid-phase in-plane growth. *Nat Energy*. **6**, 63-71 (2021).
33. Zhang, H. et al. Multimodal host–guest complexation for efficient and stable perovskite photovoltaics. *Nat Commun* **12**, 3383 (2021).
34. Pei. F. et al. Thermal Management Enables More Efficient and Stable Perovskite Solar Cells. *ACS Energy Lett.* **6**, 3029−3036 (2021).
35. Xue, J. et al. Reconfiguring the band-edge states of photovoltaic perovskites by conjugated organic cations. *Science*. **371**, 636–640 (2021).
36. Zai, H. et al. Sandwiched electrode buffer for efficient and stable perovskite solar cells with dual back surface fields. *Joule* **5**. 2148-2163 (2021).
37. Li, N. et al. Liquid medium annealing for fabricating durable perovskite solar cells with improved reproducibility. *Science*. **373**, 561-567 (2021).
38. Jung, E. H. et al. Efficient, stable and scalable perovskite solar cells using poly(3-hexylthiophene). *Nature*. **567**, 511-515 (2019).
39. Yang, Y. et al. Modulation of perovskite crystallization processes towards highly efficient and stable perovskite solar cells with MXene quantum dot-modified SnO_2. *Energy Environ. Sci.,* **14**, 3447-3454 (2021).
40. Zhu, H. et al. Synergistic Effect of Fluorinated Passivator and Hole Transport Dopant Enables Stable Perovskite Solar Cells with an Efficiency Near 24%. *J. Am. Chem. Soc.* **143**, 3231-3237 (2021).
41. Su, T.-S. et al. Crown Ether Modulation Enables over 23% Efficient Formamidinium-Based Perovskite Solar Cells. *J. Am. Chem. Soc.* **142**, 19980-19991 (2020).
42. Wang, R. et al. Constructive molecular configurations for surface-defect passivation of perovskite photovoltaics. *Science*, **366**, 1509-1513 (2019).

43. Wang, H. et al. Water Stable Haloplumbate Modulation for Efficient and Stable Hybrid Perovskite Photovoltaics. *Adv. Energy Mater*. **11**, 2101082 (2021).
44. Wang, P. et al. Cobalt Chloride Hexahydrate Assisted in Reducing Energy Loss in Perovskite Solar Cells with Record Open-Circuit Voltage of 1.20 V. *ACS Energy Lett.* **6**, 2121−2128 (2021).
45. Wang, P. Gradient Energy Alignment Engineering for Planar Perovskite Solar Cells with Efficiency Over 23%. *Adv. Mater.* **32**, 1905766 (2020).
46. Krishna, A. et al. Nanoscale interfacial engineering enables highly stable and efficient perovskite photovoltaics. *Energy Environ. Sci.* **14**, 5552-5562 (2021).
47. Yang, B. et al. Interfacial Passivation Engineering of Perovskite Solar Cells with Fill Factor over 82% and Outstanding Operational Stability on n-i-p Architecture. *ACS Energy Lett.* **6**, 3916−3923 (2021).
48. Zhang, F. et al. Metastable Dion-Jacobson 2D structure enables efficient and stable perovskite solar cells. *Science*. **375**, 71-76 (2022).
49. Peng, J. et al. Centimetre-scale perovskite solar cells with fill factors of more than 86 per cent. *Nature.* **601**, 573-578, (2022).
50. Zhao, L. et al. Enabling full-scale grain boundary mitigation in polycrystalline perovskite solids. *Sci. Adv.* **8**, eabo3733 (2022).
51. Wang, T. et al. Transporting holes stably under iodide invasion in efficient perovskite solar cells. *Science.* **377**, 1227 (2022).
52. Sidhik, S. et al. Deterministic fabrication of 3D/2D perovskite bilayer stacks for durable and efficient solar cells. *Science,* **377***,* 1425 (2022).
53. Liu, C. et al. Tuning structural isomers of phenylenediammonium to afford efficient and stable perovskite solar cells and modules. *Nat. Commun.* **12**, 6394 (2021).
54. Chen, S. et al. Stabilizing perovskite-substrate interfaces for high-performance perovskite modules. *Science*. **373**, 902-907 (2021).
55. Cao, Q. et al. Star-polymer multidentate-cross-linking strategy for superior operational stability of inverted perovskite solar cells at high efficiency. *Energy Environ. Sci*. **14**, 5406-5415 (2021).
56. Li, X. et al. Constructing heterojunctions by surface sulfidation for efficient inverted perovskite solar cells. *Science.* **375**, 434-437 (2022).
57. Li, Z. et al. Organometallic-functionalized interfaces for highly efficient inverted perovskite solar cells. *Science*. **376**, 416-420 (2022).
58. Jiang, Q. et al. Surface reaction for efficient and stable inverted perovskite solar cells. *Nature*. **611**, 278-283 (2022).
