# Supplementary material for: Multifunctional sulfonium-based treatment for perovskite solar cells with less than 1% efficiency loss over 4,500-h operational stability tests
Source: Nat Energy. 2024 Jan 4;9(2):172–83. doi: 10.1038/s41560-023-01421-6 (PMC10896729; doi:10.1038/s41560-023-01421-6)
Supplement: Supplementary file 2 — Reporting Summary [file 41560_2023_1421_MOESM2_ESM.pdf]

nature research

Corresponding author(s): Bowen Yang

Solar Cells Reporting Summary

Nature Research wishes to improve the reproducibility of the work that we publish. This form is intended for publication with all accepted papers reporting the characterization of photovoltaic devices and provides structure for consistency and transparency in reporting. Some list items might not apply to an individual manuscript, but all fields must be completed for clarity.

For further information on Nature Research policies, including our data availability policy, see Authors & Referees.

Experimental design

Please check: are the following details reported in the manuscript?

1. Dimensions

Item	Yes	No	Details
Area of the tested solar cells	☒	☐	Methods section
Method used to determine the device area	☒	☐	Methods section

2. Current-voltage characterization

Item	Yes	No	Details
Current density-voltage (J-V) plots in both forward and backward direction	☒	☐	Figure 3d
Voltage scan conditions<br>*For instance: scan direction, speed, dwell times*	☒	☐	Methods section
Test environment<br>*For instance: characterization temperature, in air or in glove box*	☒	☐	Main text and Methods section
Protocol for preconditioning of the device before its characterization	☐	☒	No preconditioning was involved before test
Stability of the J-V characteristic<br>*Verified with time evolution of the maximum power point or with the photocurrent at maximum power point; see ref. 7 for details.*	☒	☐	Figure 3e, Figure 4a, 4c-4e

3. Hysteresis or any other unusual behaviour

Item	Yes	No	Details
Description of the unusual behaviour observed during the characterization	☐	☒	No unusual behavior was observed
Related experimental data	☐	☒	No unusual behavior was observed

4. Efficiency

Item	Yes	No	Details
External quantum efficiency (EQE) or incident photons to current efficiency (IPCE)	☒	☐	Supplementary Figure 21
A comparison between the integrated response under the standard reference spectrum and the response measure under the simulator	☒	☐	Characterization section
For tandem solar cells, the bias illumination and bias voltage used for each subcell	☐	☒	No tandem solar cells were reported in our manuscript

5. Calibration

Item	Yes	No	Details
Light source and reference cell or sensor used for the characterization	☒	☐	Methods section
Confirmation that the reference cell was calibrated and certified	☒	☐	Methods section

Calculation of spectral mismatch between the reference cell and the devices under test

☐ Yes
☒ No

The light spectrum used for measurements matches well with the reference cell and AM1.5.

6. Mask/aperture

Size of the mask/aperture used during testing

☒ Yes
☐ No

Methods section

Variation of the measured short-circuit current density with the mask/aperture area

☐ Yes
☒ No

We measured all devices with identical mask

7. Performance certification

Identity of the independent certification laboratory that confirmed the photovoltaic performance

☐ Yes
☒ No

No new record efficiency is claimed. The focus of our manuscript is on the long-term stability of the devices.

A copy of any certificate(s)
Provide in Supplementary Information

☐ Yes
☒ No

No new record efficiency is claimed. The focus of our manuscript is on the long-term stability of the devices.

8. Statistics

Number of solar cells tested

☒ Yes
☐ No

Supplementary Figure 19, Figure 4a, 4c and 4d, Supplementary Table 7, Supplementary 25a, Supplementary Figure 29

Statistical analysis of the device performance

☒ Yes
☐ No

Main text, Supplementary Figure 19, Figure 4a, 4c and 4d

9. Long-term stability analysis

Type of analysis, bias conditions and environmental conditions
For instance: illumination type, temperature, atmosphere humidity, encapsulation method, preconditioning temperature

☒ Yes
☐ No

Main text, Methods section
