# Supplementary figures and images for: Multifunctional sulfonium-based treatment for perovskite solar cells with less than 1% efficiency loss over 4,500-h operational stability tests
Source: Nat Energy. 2024 Jan 4;9(2):172–83. doi: 10.1038/s41560-023-01421-6 (PMC10896729; doi:10.1038/s41560-023-01421-6)

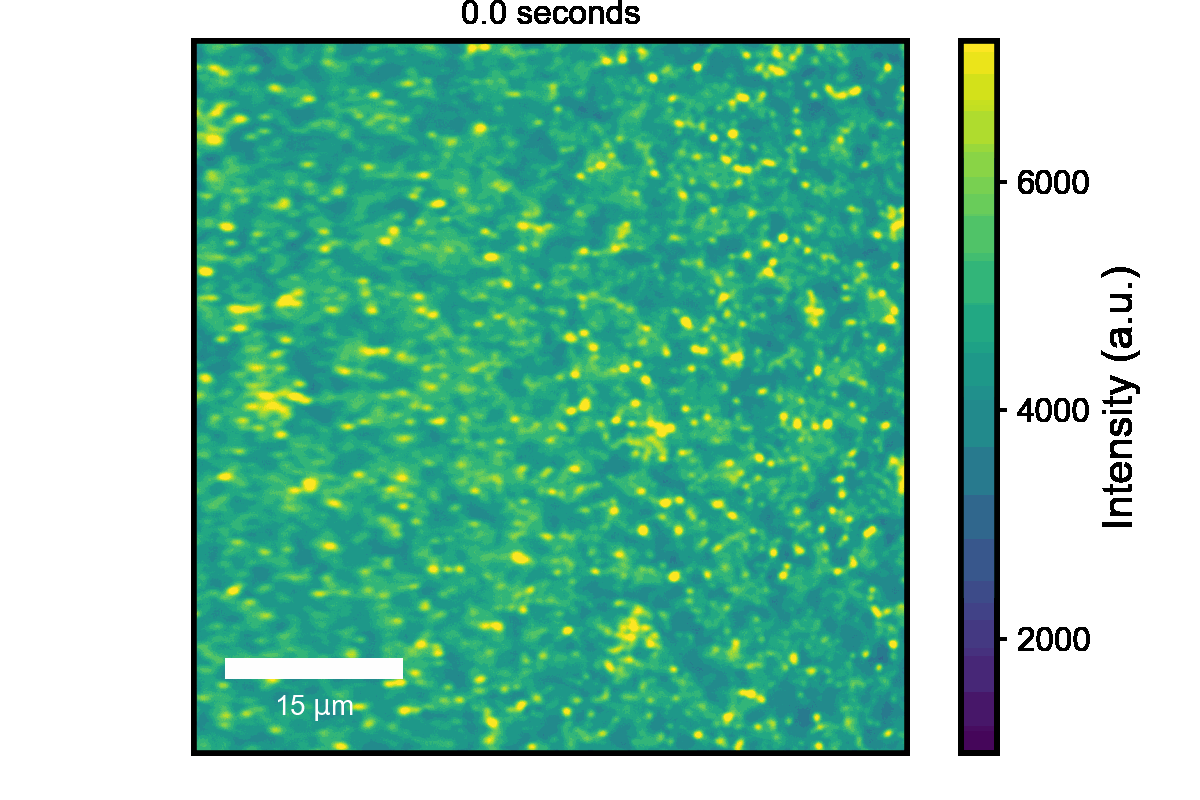

Supplement: Supplementary file 3 — Hyperspectral photoluminescence mapping of reference film. [file 41560_2023_1421_MOESM3_ESM.gif]

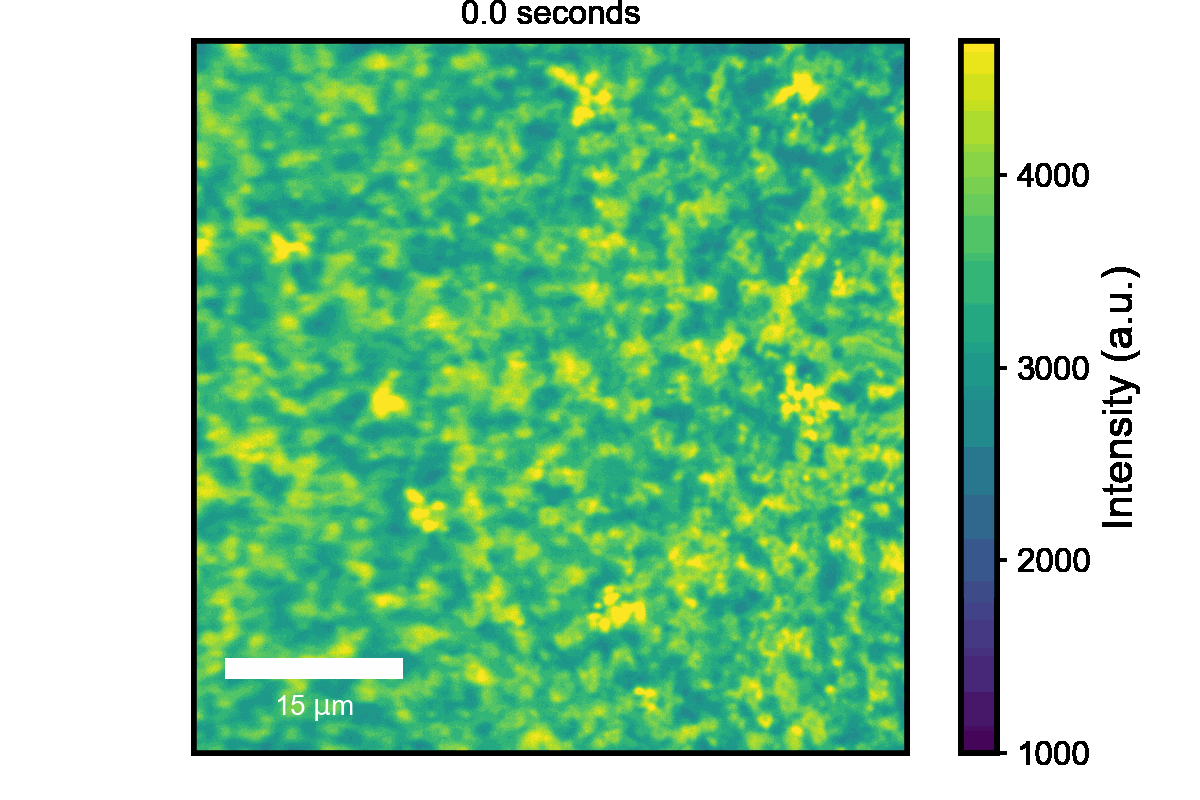

Supplement: Supplementary file 4 — Hyperspectral photoluminescence mapping of DMPESI-treated film. [file 41560_2023_1421_MOESM4_ESM.gif]
